# Supplementary material for: The DNA repair protein DNA-PKcs modulates synaptic plasticity via PSD-95 phosphorylation and stability
Source: EMBO Rep. 2024 Jul 31;25(8):27. doi: 10.1038/s44319-024-00198-3 (PMC11315936; doi:10.1038/s44319-024-00198-3)
Supplement: Supplementary file 1 — Appendix [file 44319_2024_198_MOESM1_ESM.pdf]

Appendix for Article:

The DNA repair protein DNA-PKcs modulates synaptic plasticity via PSD-95 phosphorylation and stability

Cristiana Mollinari,[1,2]* Alessio Cardinale,[3] * Leonardo Lupacchini,[4]* Alberto Martire,[5] Valentina Chiodi,[5] Andrea Martinelli,[6] Anna Maria Rinaldi,[7] Massimo Fini,[4] Simonetta Pazzaglia,[8] Maria Rosaria Domenici,[5] Enrico Garaci,[4,9] Daniela Merlo[1§]

* Equal contribution
§ Corresponding author

Table of Contents

- Appendix Figure S1 page 3
- Appendix Figure S2 page 4
- Appendix Figure S3 page 5
- Appendix Figure S4 page 6
- Appendix Figure S5 page 7
- Appendix Figure S6 page 8
- Appendix Figure S7 page 9
- Appendix Figure S8 page 10
- Appendix Figure S9 page 11
- Appendix Figure S10 page 12
- Appendix Materials and Methods page 13
- Appendix References page 14

Appendix Figure S1

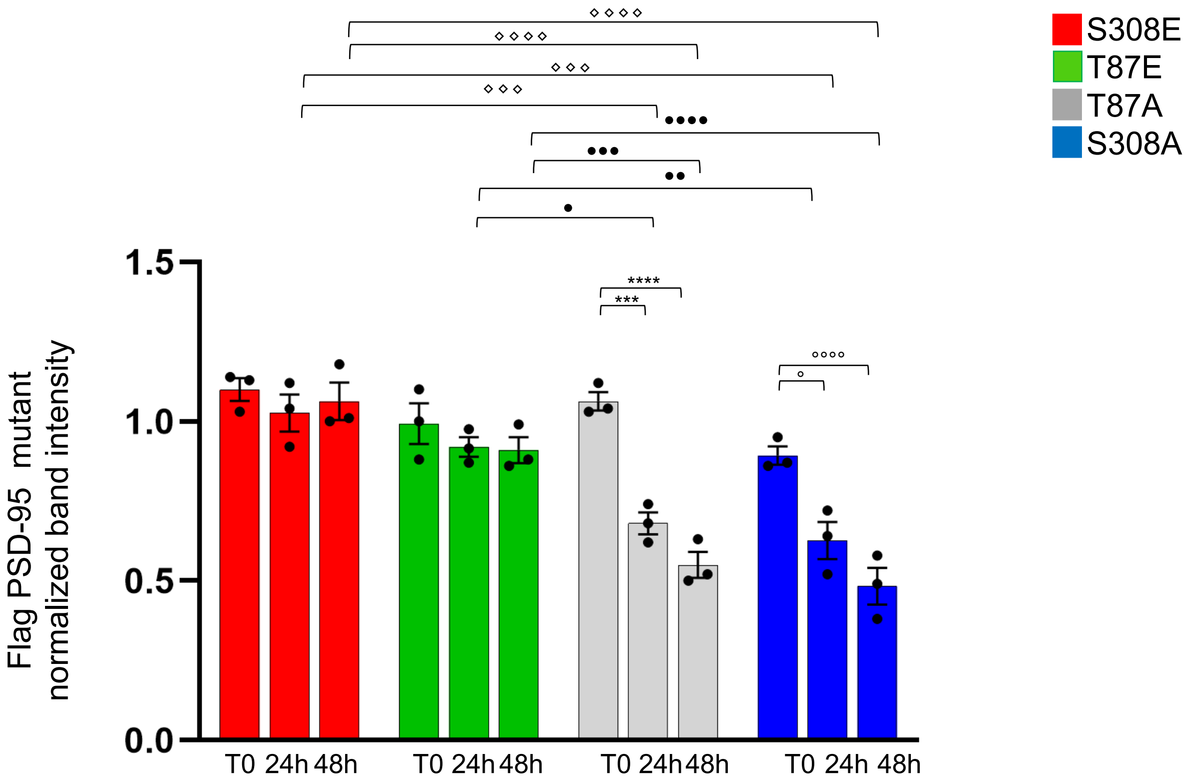

Appendix Figure S1. PSD-95S308E and PSD-95T87E mutants over-expressed in WT neurons remain stable whereas PSD-95T87A and PSD-95S308A decrease over time in WT neurons.

Values in the plot represent quantification of PSD-95S308E, PSD-95T87E, PSD-95T87A and PSD-95S308A mutant protein levels over time following cycloheximide treatment and normalized to α-Tubulin. (means ± SEM; n = 3). Statistics by two-way ANOVA followed by Tukey's Post Hoc Analysis. ⋄⋄⋄⋄p < 0.0001 S308E 48 h vs S308A 48 h; ⋄⋄⋄⋄ p < 0.0001 S308E 48 h vs T87A 48 h; ⋄⋄⋄ p < 0.001 S308E 24 h vs S308A 24h; ⋄⋄⋄ p < 0.001 S308E 24 h vs T87A 24h; •••• p < 0.0001 T87E 48 h vs S308A 48 h; ••• p< 0.001 T87E 48 h vs T87A 48 h; •• p < 0.005 T87E 24 h vs S308 24 h; • p < 0.05 T87E 24 h vs T87A 24 h; ****p < 0.0001 T87A 48 h vs T0; ***p < 0.001 T87A 24 h vs T0; °°°°p < 0.0001 S308A 48 h vs T0; °p < 0.05 S308A 24 h vs T0.

Appendix Figure S2

A

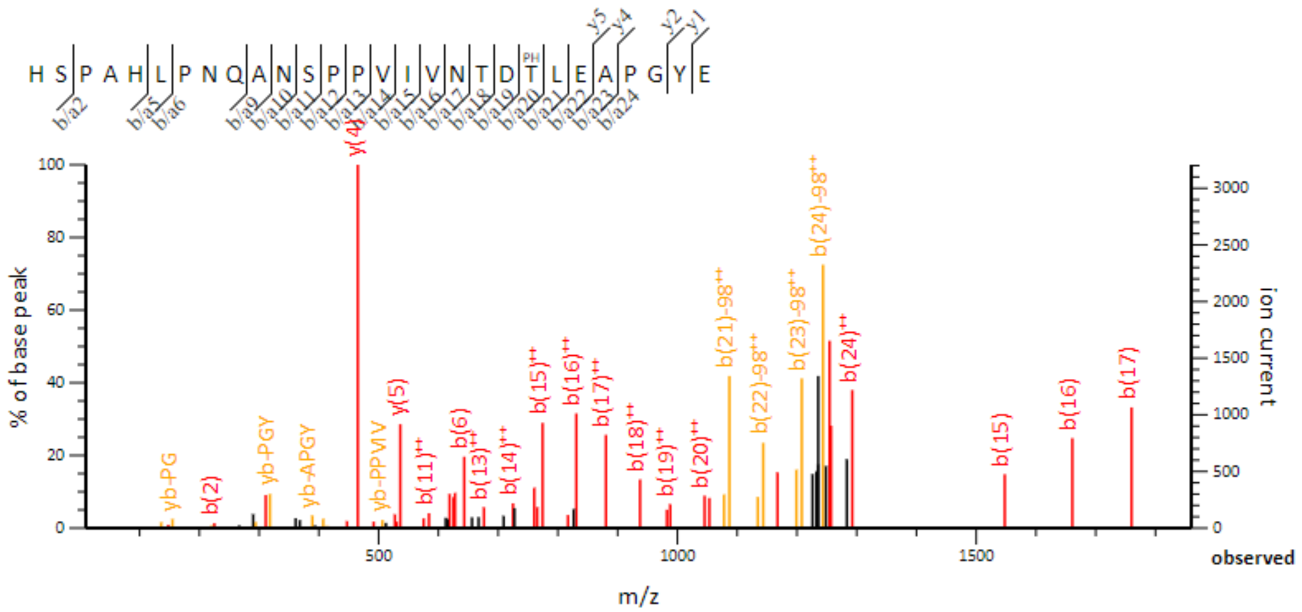

B

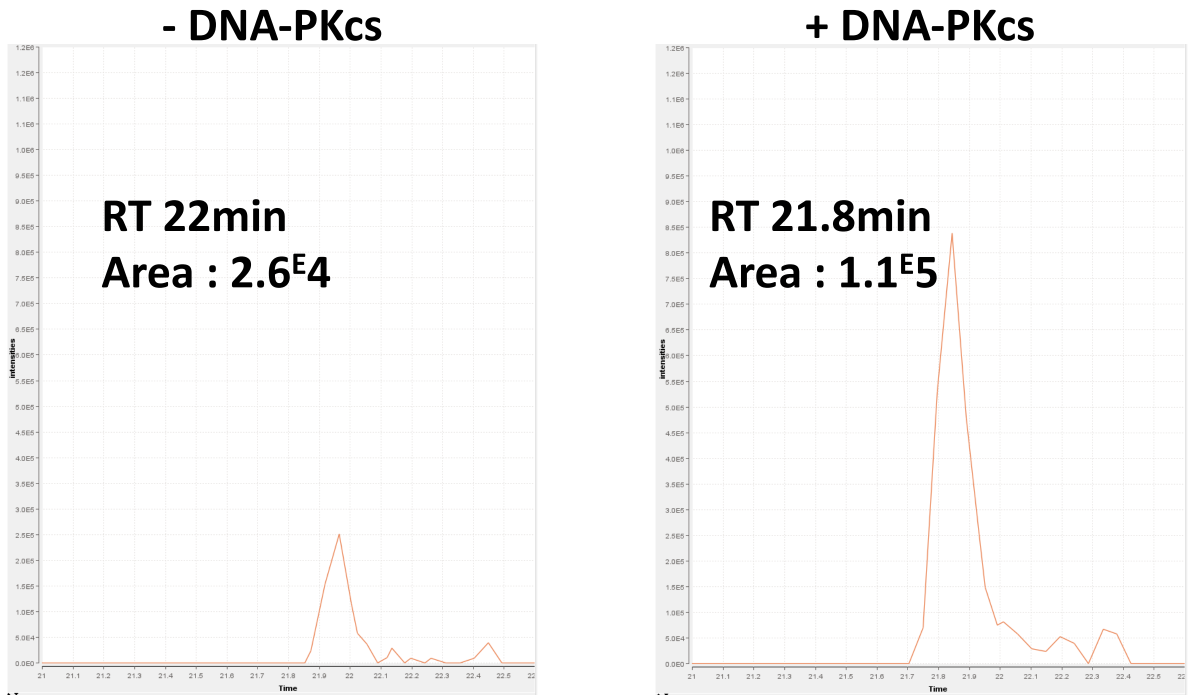

Appendix Figure S2. LC-MS/MS analysis of peptide HSPAHLPNQANSPPVIVNTD**T**LEAPGYE from DLG4 phosphorylated on T87. This peptide was identified with 4 MS/MS spectra.

A Best HCD spectrum from the precursor ion 1016.8078Th in the 3+ charge state obtained at 21.8min. This spectrum was scored 147 by Mascot and confidence on phosphosite localization was 99.6%. Fragment ions matching with the theoritical spectrum are annotated.

B Extracted ion chromatogram of m/z 1016.8078Th in samples without kinase or with DNA-PKcs, digested by GluC.

Appendix Figure S3

A

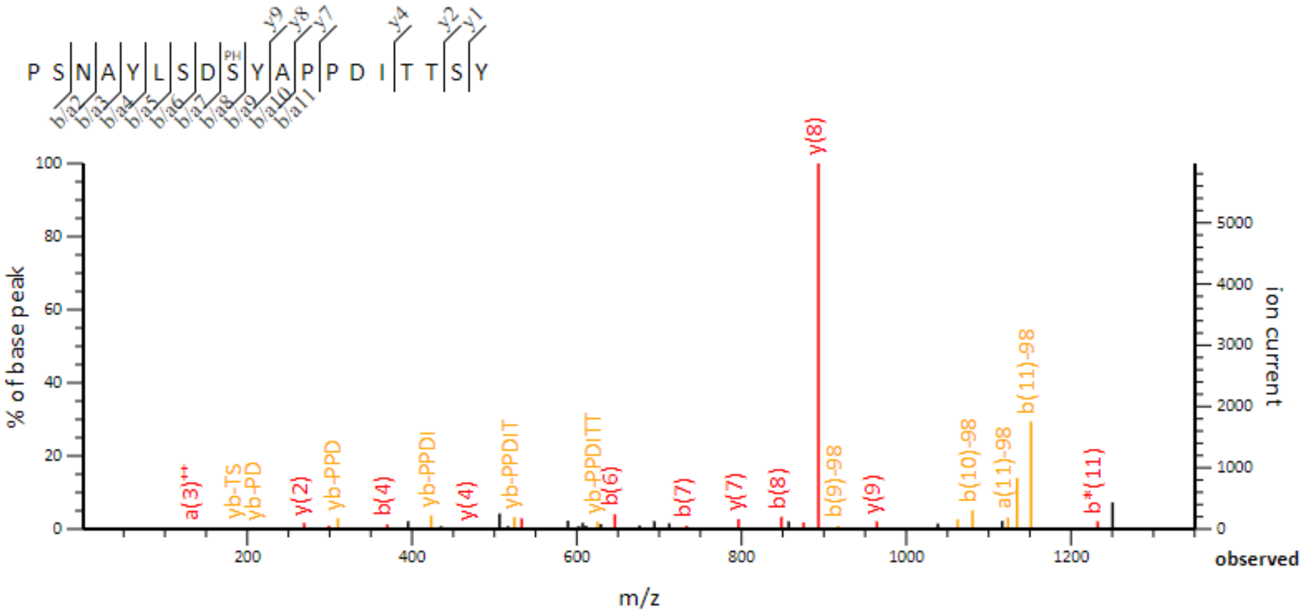

B

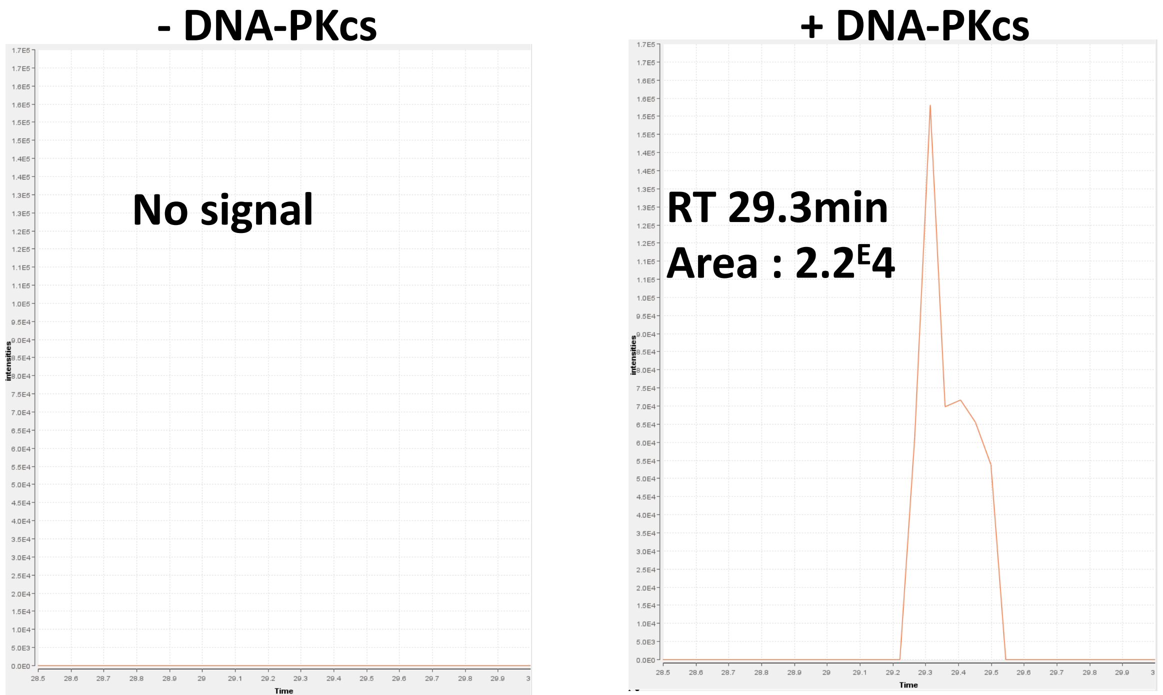

Appendix Figure S3. LC-MS/MS analysis of peptide PSNAYLSD**S**YAPPDITTSY from DLG4 phosphorylated on S297. This peptide was identified once.

A HCD spectrum from the precursor ion 1071.4503Th in the 2+ charge state obtained at 29.3min. This spectrum was scored 74 by Mascot and confidence on phosphosite localization was 97.6%. Fragment ions matching with the theoritical spectrum are annotated.

B Extracted ion chromatogram of m/z 1071.4503Th in samples without kinase or with DNA-PKcs, digested by the mix of Chymotrypsin and Trypsin

Appendix Figure S4

A

B

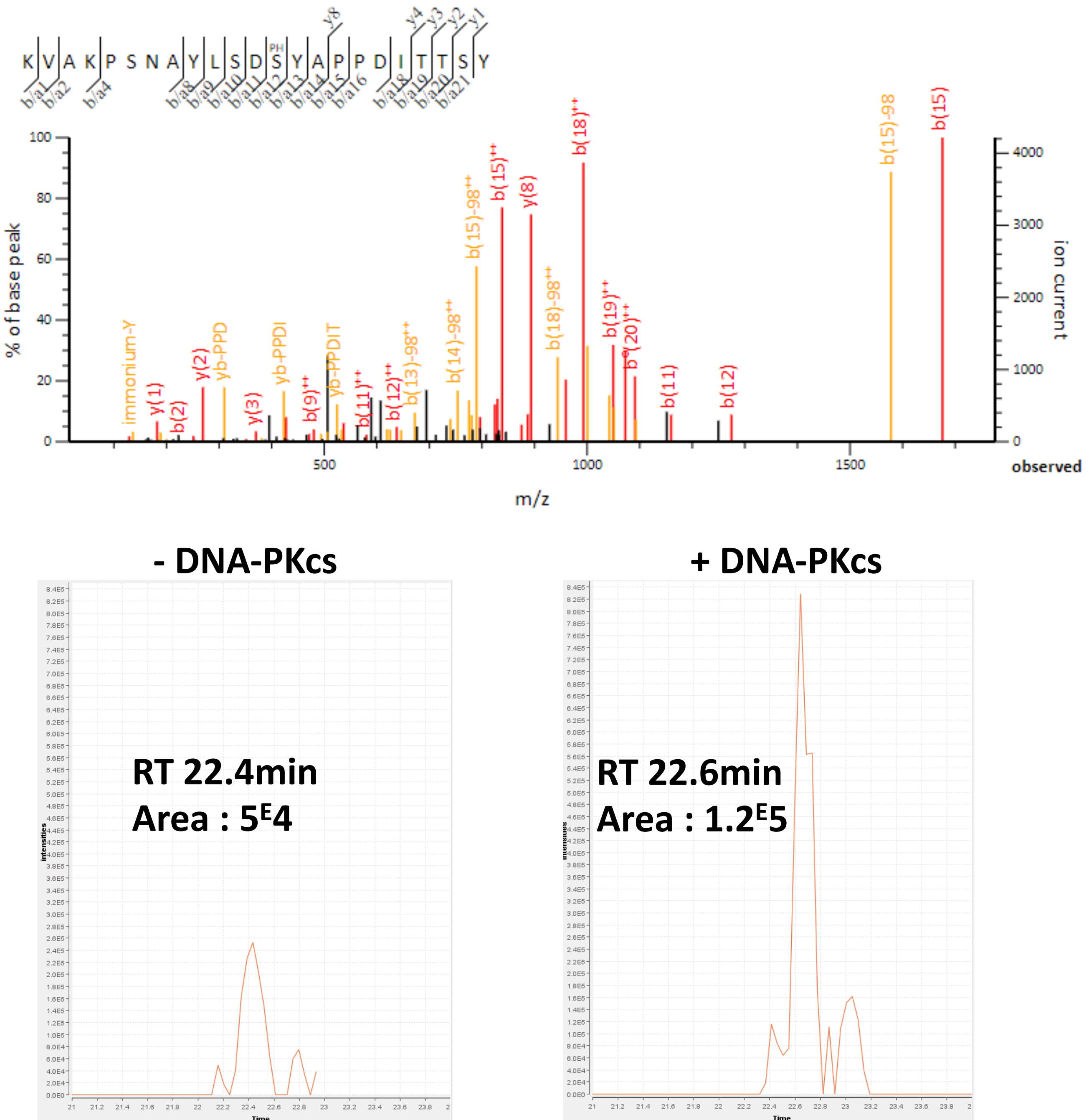

Appendix Figure S4. LC-MS/MS analysis of peptide KVAKPSNAYLSD**S**YAPPDITTSY from DLG4 phosphorylated on S297. This peptide was identified with 4 spectra.

A Best HCD spectrum from the precursor ion 856.7350Th in the 3+ charge state obtained at 22.7min. This spectrum was scored 61 by Mascot and confidence on phosphosite localization was 94.8%. Fragment ions matching with the theoritical spectrum are annotated.

B Extracted ion chromatogram of m/z 856.7350Th in samples without kinase or with DNA-PKcs, digested by Chymotrypsin

Appendix Figure S5

A

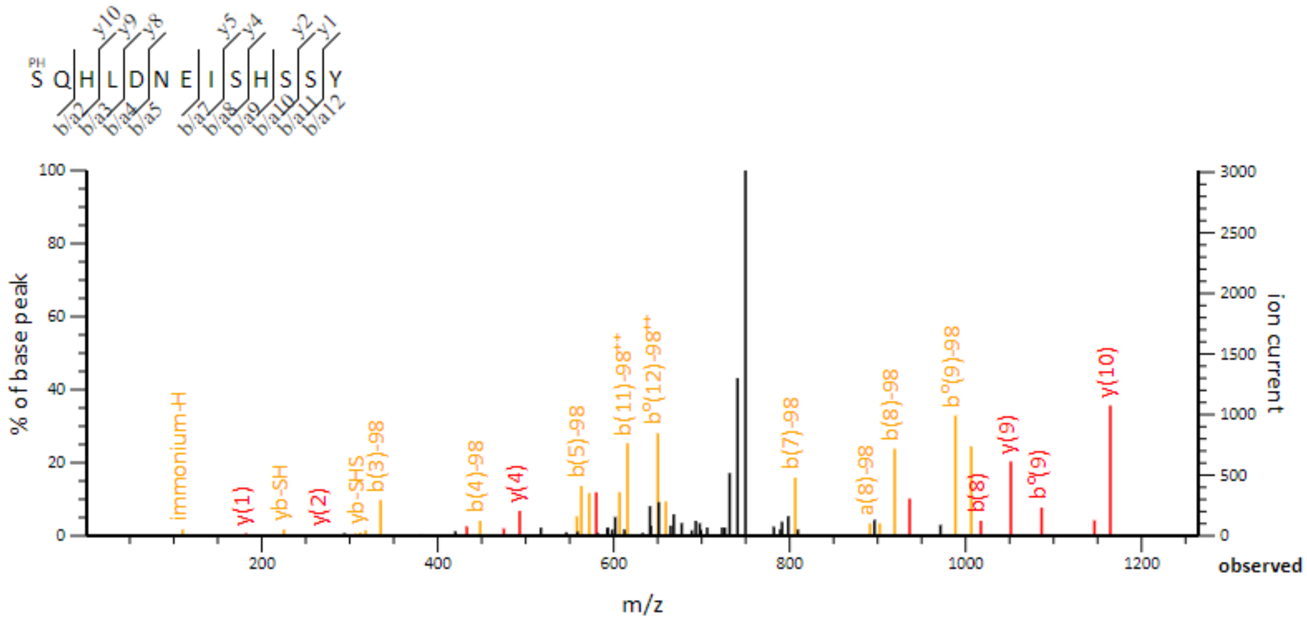

B

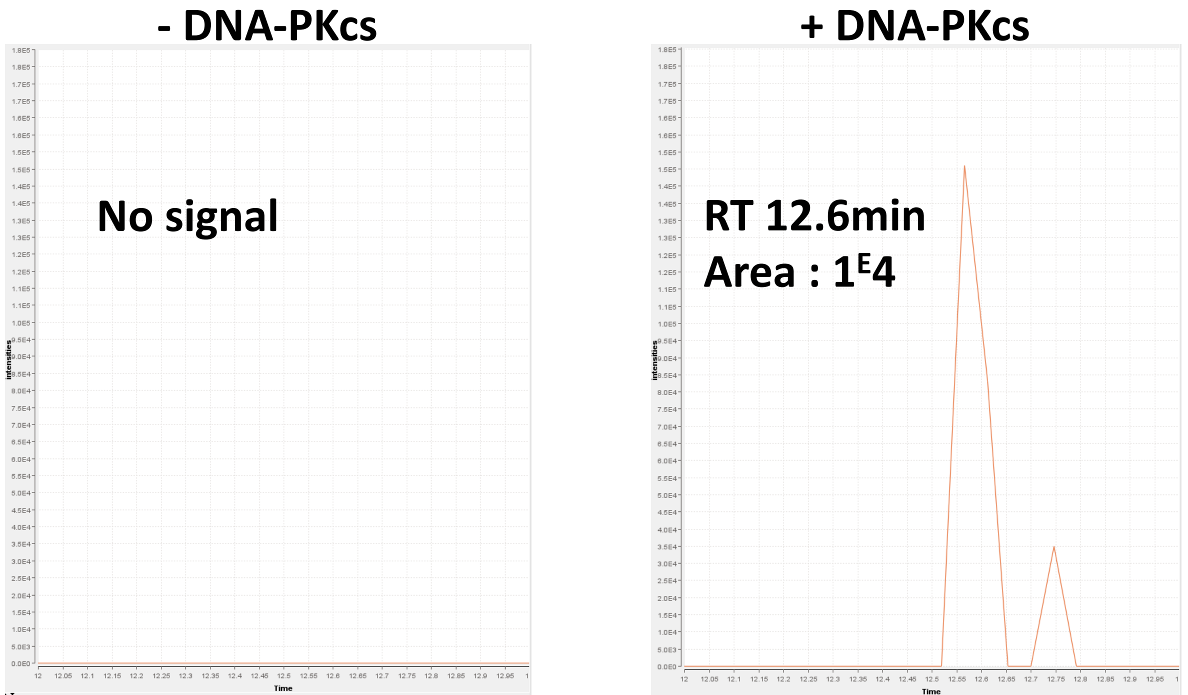

Appendix Figure S5. LC-MS/MS analysis of peptide **S**QHLDNEISHSSY from DLG4 phosphorylated on S308. This peptide was identified once.

A HCD spectrum from the precursor ion 798.8198 Th in the 2+ charge state obtained at 12.6min. This spectrum was scored 60 by Mascot and confidence on phosphosite localization was 100%. Fragment ions matching with the theoritical spectrum are annotated.

B Extracted ion chromatogram of m/z 798.8198 in samples without kinase or with DNA-PKcs, digested by Chymotrypsin.

Appendix Figure S6

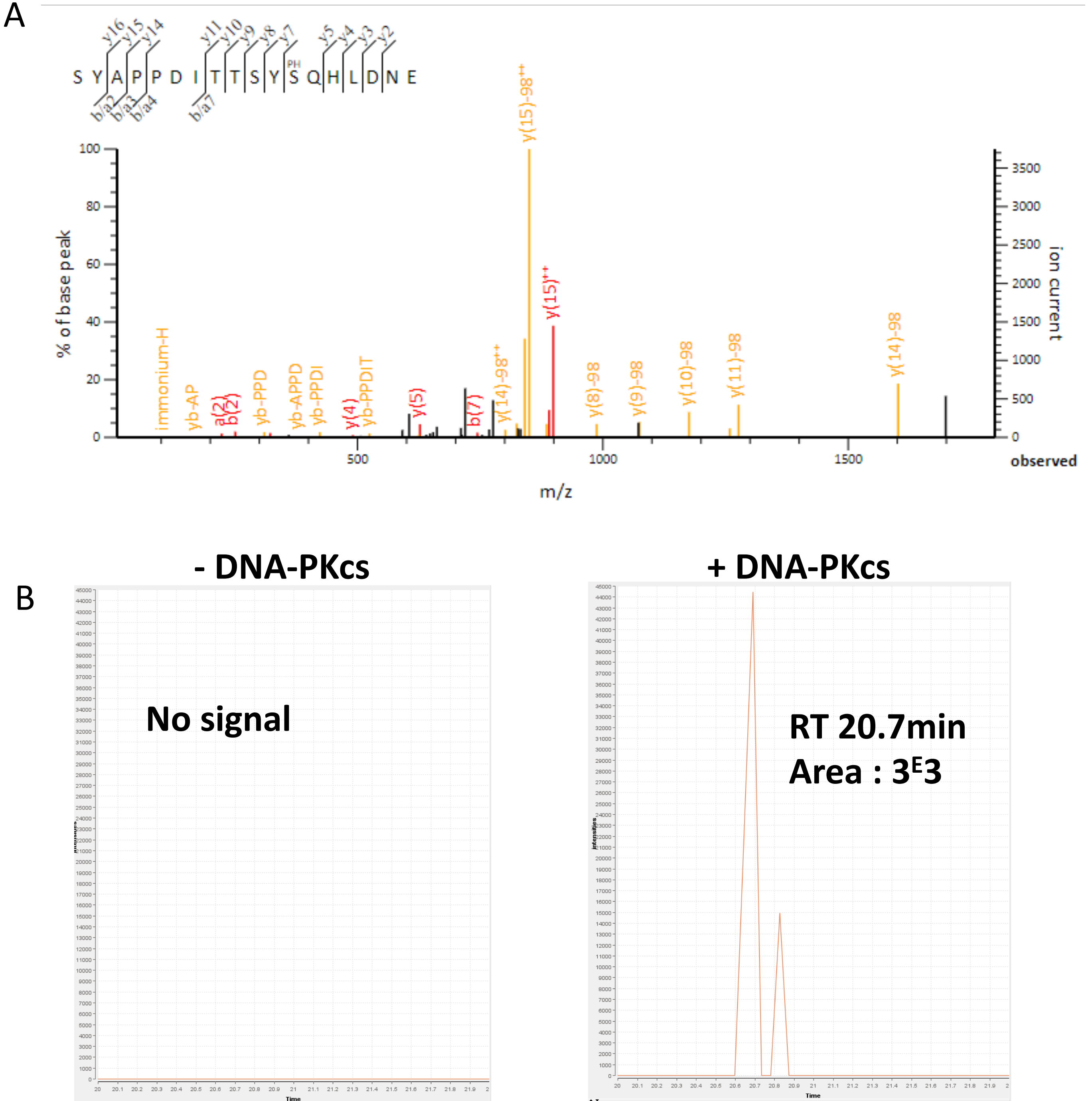

Appendix Figure S6. LC-MS/MS analysis of peptide SYAPPDITTSYSQHLDNE from DLG4 phosphorylated on S308. This peptide was identified once.

A HCD spectrum from the precursor ion 1059.4399 Th in the 2+ charge state obtained at 20.7min. This spectrum was scored 53 by Mascot and confidence on phosphosite localization was 97.6%. Fragment ions matching with the theoritical spectrum are annotated.

B Extracted ion chromatogram of m/z 1059.4399 Th in samples without kinase or with DNA-PKcs, digested by GluC.

Appendix Figure S7

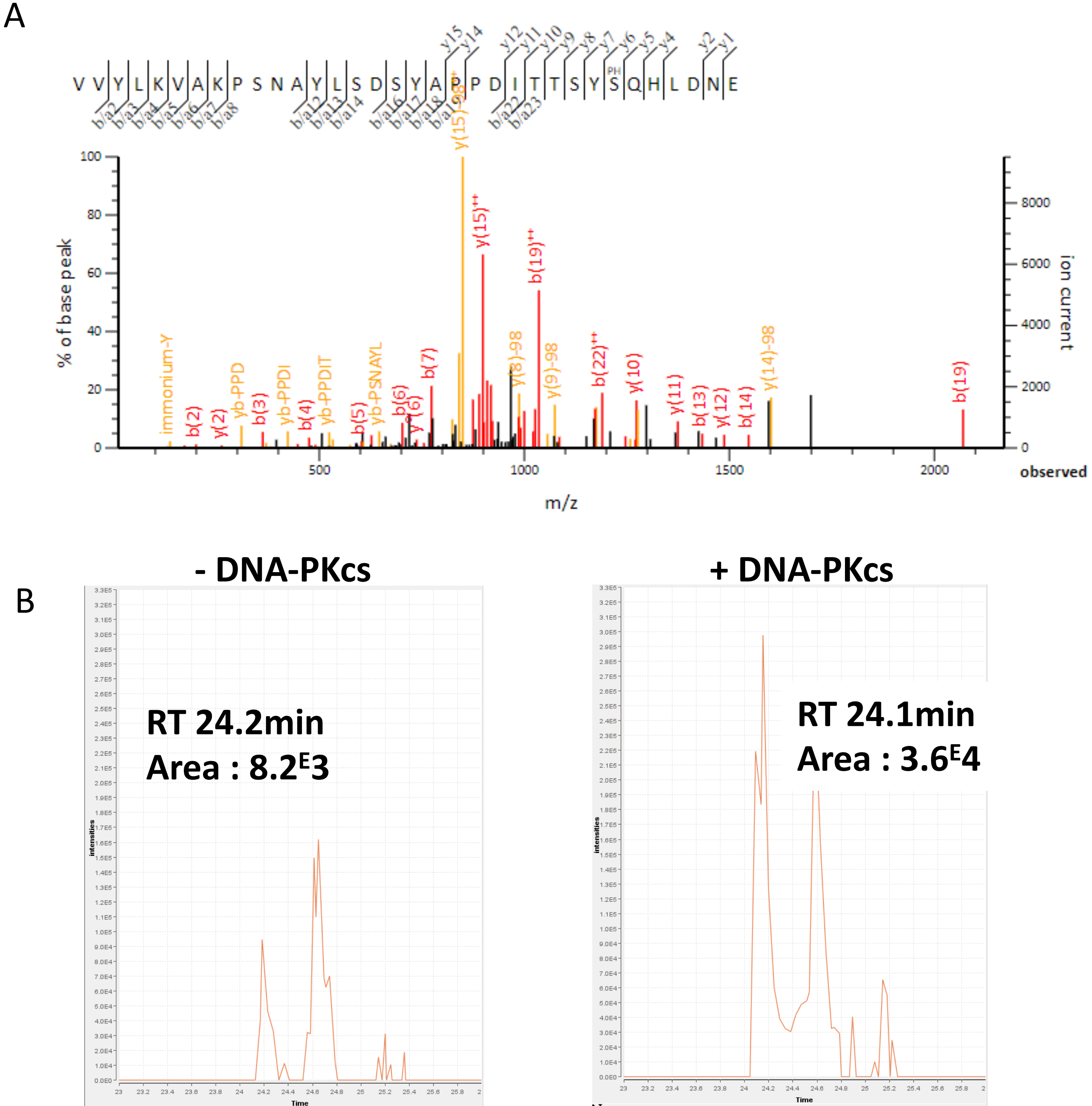

Appendix Figure S7. LC-MS/MS analysis of peptide VVYLKVAKPSNAYLSDSYAPPDITTSY**S**QHLDNE from DLG4 phosphorylated on S308. This peptide was identified once.

A HCD spectrum from the precursor ion 967.2108Th in the 4+ charge state obtained at 24.1min. This spectrum was scored 84 by Mascot and confidence on phosphosite localization was 92.8%. Fragment ions matching with the theoritical spectrum are annotated.

B Extracted ion chromatogram of m/z 967.2108Th in samples without kinase or with DNA-PKcs, digested by GluC.

Appendix Figure S8

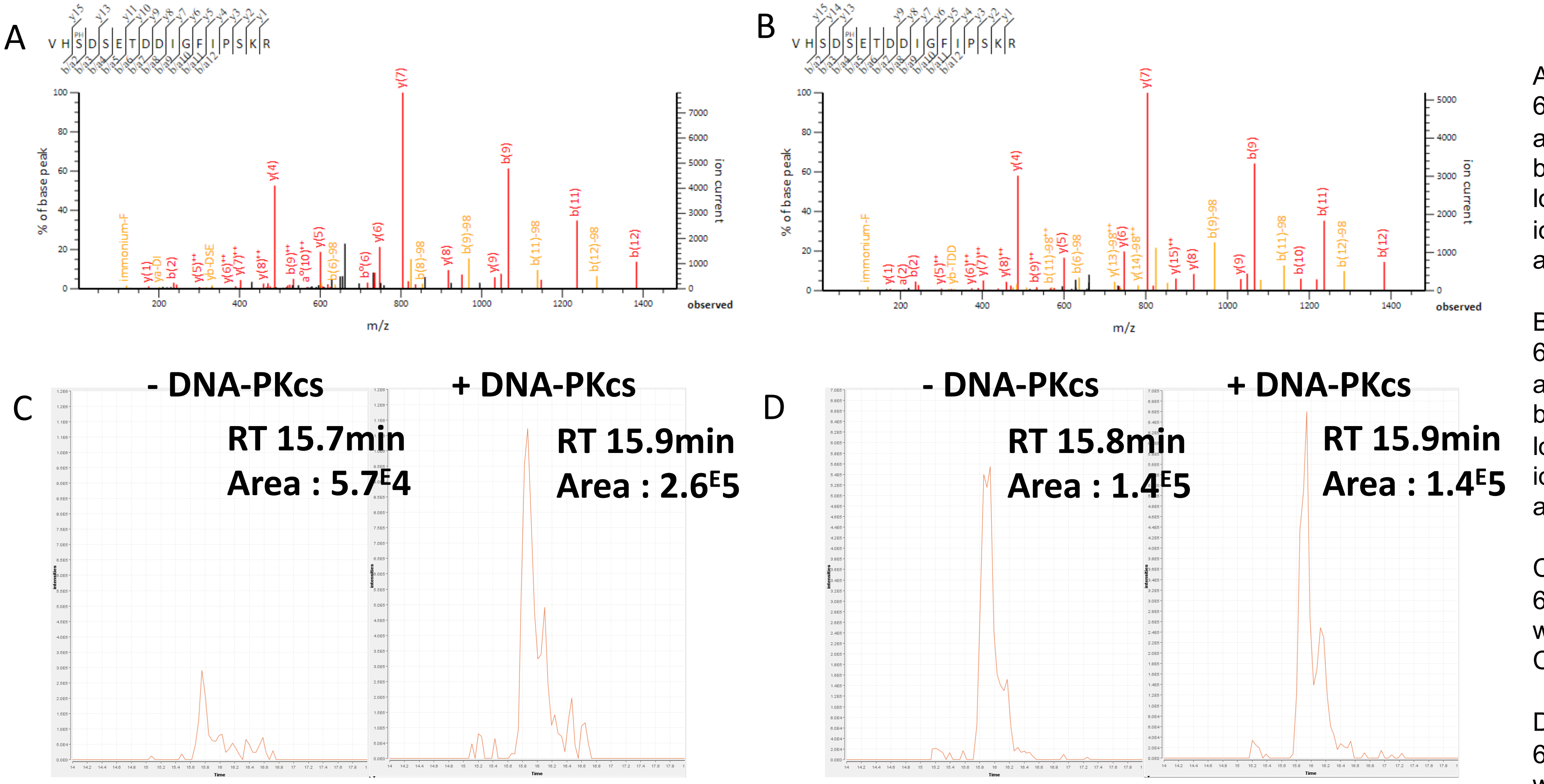

Appendix Figure S8. LC-MS/MS analysis of peptide VHSDSETDDIGFIPSKR and VHSDSETDDIGFIPSKR from DLG4 phosphorylated on S521 or S523. This peptide was identified with 3 spectra confirming coexistence of both position.

A HCD spectrum from the precursor ion 661.6323Th in the 3+ charge state obtained at 15.9min. This spectrum was scored 80 by Mascot and confidence on phosphosite localization S521 was 86.7%. Fragment ions matching with the theoritical spectrum are annotated.

B HCD spectrum from the precursor ion 661.6323Th in the 3+ charge state obtained at 15.8min. This spectrum was scored 83 by Mascot and confidence on phosphosite localization S523 was 95.2%. Fragment ions matching with the theoritical spectrum are annotated.

C Extracted ion chromatogram of m/z 661.6323Th in samples without kinase or with DNA-PKcs, digested by the mix of Chymotrypsin and Trypsin

D Extracted ion chromatogram of m/z 661.6323Th in samples without kinase or with DNA-PKcs, digested by Trypsin

Appendix Figure S9

A
B

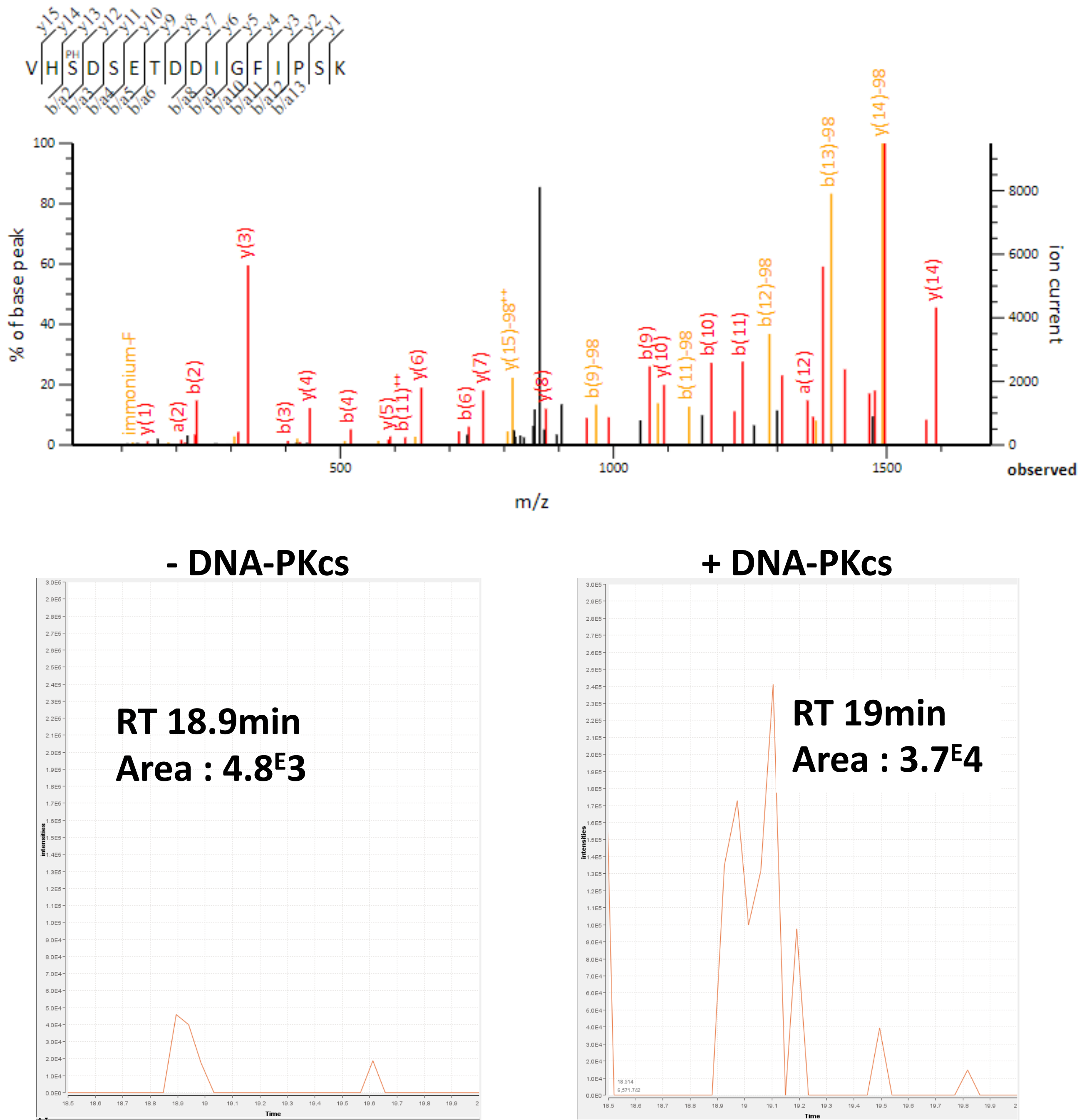

Appendix Figure S9. LC-MS/MS analysis of peptide VHSDSETDDIGFIPSK from DLG4 phosphorylated on S521. This peptide was identified twice.

A HCD spectrum from the precursor ion 913.8975Th in the 2+ charge state obtained at 19.1min. This spectrum was scored 135 by Mascot and confidence on phosphosite localization was 99.9%. Fragment ions matching with the theoritical spectrum are annotated.

B Extracted ion chromatogram of m/z 913.8975Th in samples without kinase or with DNA-PKcs, digested by the mix of Chymotrypsin and Trypsin

Appendix Figure S10

A

B

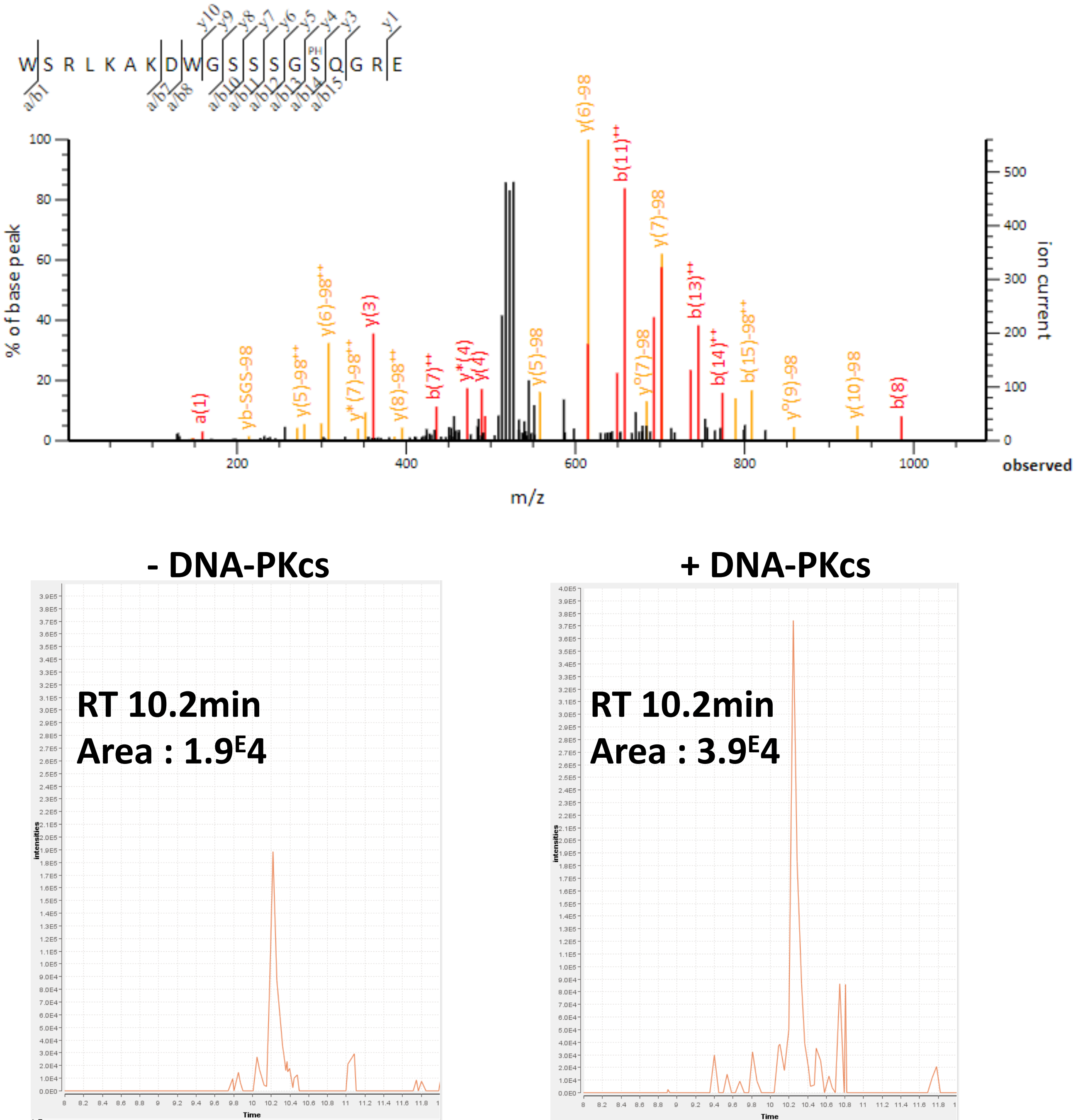

Appendix Figure S10. LC-MS/MS analysis of peptide WSRLKAKDWGSSSG**S**QGRE from DLG4 phosphorylated on S556. This peptide was identified twice.

A HCD spectrum from the precursor ion 551.2579Th in the 4+ charge state obtained at 10.2min. This spectrum was scored 62 by Mascot and confidence on phosphosite localization was 98%. Fragment ions matching with the theoritical spectrum are annotated.

B Extracted ion chromatogram of m/z 551.2579Th in samples without kinase or with DNA-PKcs, digested by GluC.

Appedix Materials and Methods

Mass Spectrometry-Based Proteomic Analyses

Two samples were provided: 10μg DLG4 without kinase (100μL) and 10μg DLG4 with DNA-PKcs (100μL) for nano-LC-MS analysis. The samples were solubilized in Laemmli buffer before stacking of proteins in the top layer of a 4–12% NuPAGE gel (Invitrogen) for separation followed by R-250 Coomassie blue staining. The gel bands were manually excised and digested as previously described (Salvetti *et al*, 2016) using either modified trypsin, chymotrypsin, GluC in phosphate buffer or a mix of trypsin and chymotrypsin (all sequencing grade, Promega). After peptide extraction, the samples were split into two parts before drying: ten percent for proteome analysis and ninety percent for phosphopeptide enrichment. The phosphopeptides were enriched with TiO_2 beads (GL Science) in batch mode with a modified protocol from (Jensen & Larsen, 2007). Briefly, the samples were mixed for 1h with 0.6 mg beads in loading buffer (1M glycolic acid in 80% acetonitrile (v/v) and 5% TFA (v/v)). The beads were washed three times with loading buffer, 80% acetonitrile with 1% TFA (v/v), and finally 10% acetonitrile (v/v) with 0.1% TFA (v/v). The phosphopeptides were eluted with 10% ammonia solution (v/v) for 10 min. After acidification with formic acid, the peptides were desalted using C18 ultra-micro spin columns (Harvard Apparatus) and dried under vacuum. The dried extracted peptides were resuspended in 5% acetonitrile and 0.1% trifluoroacetic acid and analyzed by online nano-liquid chromatography coupled to tandem mass spectrometry (LC–MS/MS) (Ultimate 3000 RSLCnano and the Q-Exactive Plus, Thermo Fisher Scientific). The peptides were sampled on a 300-μm internal diameter, 5-mm length PepMap C18 precolumn (Thermo Fisher Scientific) and separated on a 75-μm internal diameter, 250-mm length C18 column (Reprosil-Pur 120 C18-AQ, 1.9 μm particles, Dr. Maisch HPLC GmbH). The column flow rate was 300 nL/min. The mobile phases consisted of solution A (water with 0.1% (v/v) formic acid) and solution B (acetonitrile with 0.1% (v/v) formic acid). The peptides were eluted with a gradient consisting of an increase in solvent B from 5 to 13% in 1 min, then from 13 to 31% over 25 min, and from 31 to 41% over 4 min. For data-dependent acquisition (DDA), the spray voltage was set at 1.5 kV and the heated capillary was adjusted to 250°C. Survey full-scan MS spectra (m/z = 350–1,600) were acquired with a resolution of 140,000 after the accumulation of 3×10^6 ions (maximum filling time 100 ms). The 10 most intense ions were fragmented by higher-energy collisional dissociation (HCD) after the accumulation of 5×10^5 ions (maximum filling time 200 ms). MS and MS/MS data were acquired using the software Xcalibur (Thermo Scientific).

Mass Spectrometry-Based Proteomic Data Processing

The data were processed automatically using Mascot Distiller software (version 2.7.1.0, Matrix Science). The peptides and proteins were identified using Mascot (version 2.8) through concomitant searches against Uniprot (Homo sapiens taxonomy, 2023_01, release), a database of 250 classical contaminants (homemade) and a database with recombinant DLG4 sequence. For each different sample, the correct enzyme was chosen and set as semi-specific with three missed cleavages allowed. Precursor and fragment mass error tolerances were set, respectively, to 10 and 20 ppm. Peptide modifications allowed during the search were cysteine carbamidomethylation (fixed), acetyl (protein N-terminal, variable), methionine oxidation (variable), and serine, threonine, tyrosine phosphorylation (variable). Proline software (version 2.2) (Bouyssie *et al*, 2020) was used to merge DDA results from proteome analysis and phosphopeptides enrichment. After combination, the results were filtered: conservation of rank 1 peptide-spectrum matches (PSMs) with a minimal length of 6 amino acids and a minimal score of 25. A minimum of two peptides with one specific peptide per identified protein group was required. Proline was then used to perform MS1-based label-free quantification of the peptides and protein groups. Quantification was performed separately on proteome runs and enriched phosphopeptides runs. Cross-assignment was only activated between runs from samples obtained with the same enzyme. Protein abundances were computed as a sum of specific peptide abundances without using phosphopeptides and their counterparts. DLG4 phosphoclusters were quantified and normalized by DLG4 protein abundance. Clusters were further selected for manual curation if the confidence on the phosphosite localization was higher than 0.75, and if a differential abundance was observed after phosphopeptides enrichment (observed only with kinase or with a log2 (fold change) higher than 1.5). Their abundance was then measured in the proteome runs.

Appendix References

Bouyssie D, Hesse AM, Mouton-Barbosa E, Rompais M, Macron C, Carapito C, Gonzalez de Peredo A, Coute Y, Dupierris V, Burel A *et al* (2020) Proline: an efficient and user-friendly software suite for large-scale proteomics. *Bioinformatics* 36: 3148-3155

Jensen SS, Larsen MR (2007) Evaluation of the impact of some experimental procedures on different phosphopeptide enrichment techniques. *Rapid Commun Mass Spectrom* 21: 3635-3645

Salvetti A, Coute Y, Epstein A, Arata L, Kraut A, Navratil V, Bouvet P, Greco A (2016) Nuclear Functions of Nucleolin through Global Proteomics and Interactomic Approaches. *J Proteome Res* 15: 1659-1669
