# Supplementary figures and images for: The DNA repair protein DNA-PKcs modulates synaptic plasticity via PSD-95 phosphorylation and stability
Source: EMBO Rep. 2024 Jul 31;25(8):27. doi: 10.1038/s44319-024-00198-3 (PMC11315936; doi:10.1038/s44319-024-00198-3)

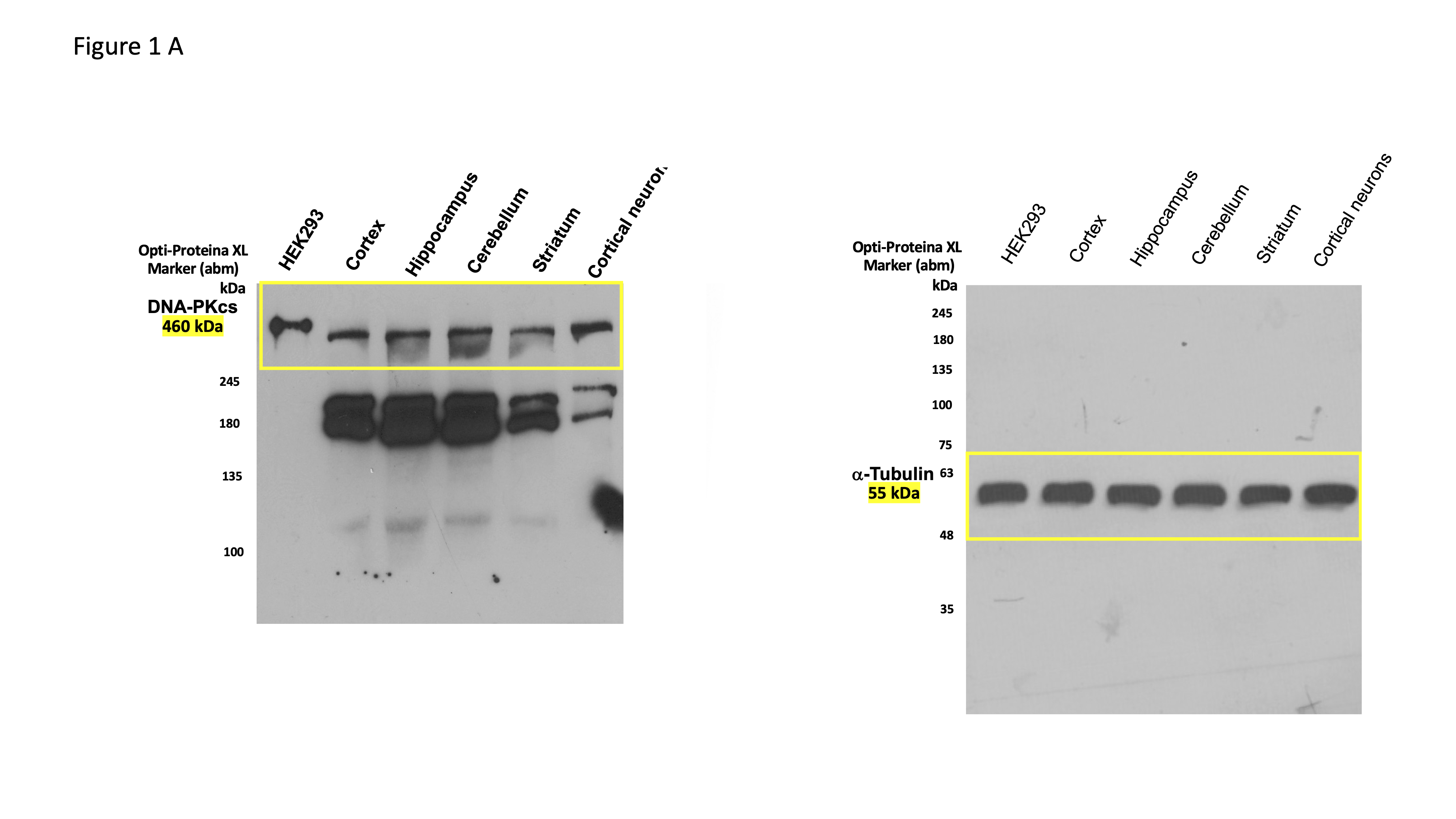

Supplement: Supplementary file 4 — Source data Fig. 1 [file 44319_2024_198_MOESM4_ESM.zip › Figure 1/Figure 1A/Figure 1A.tiff]

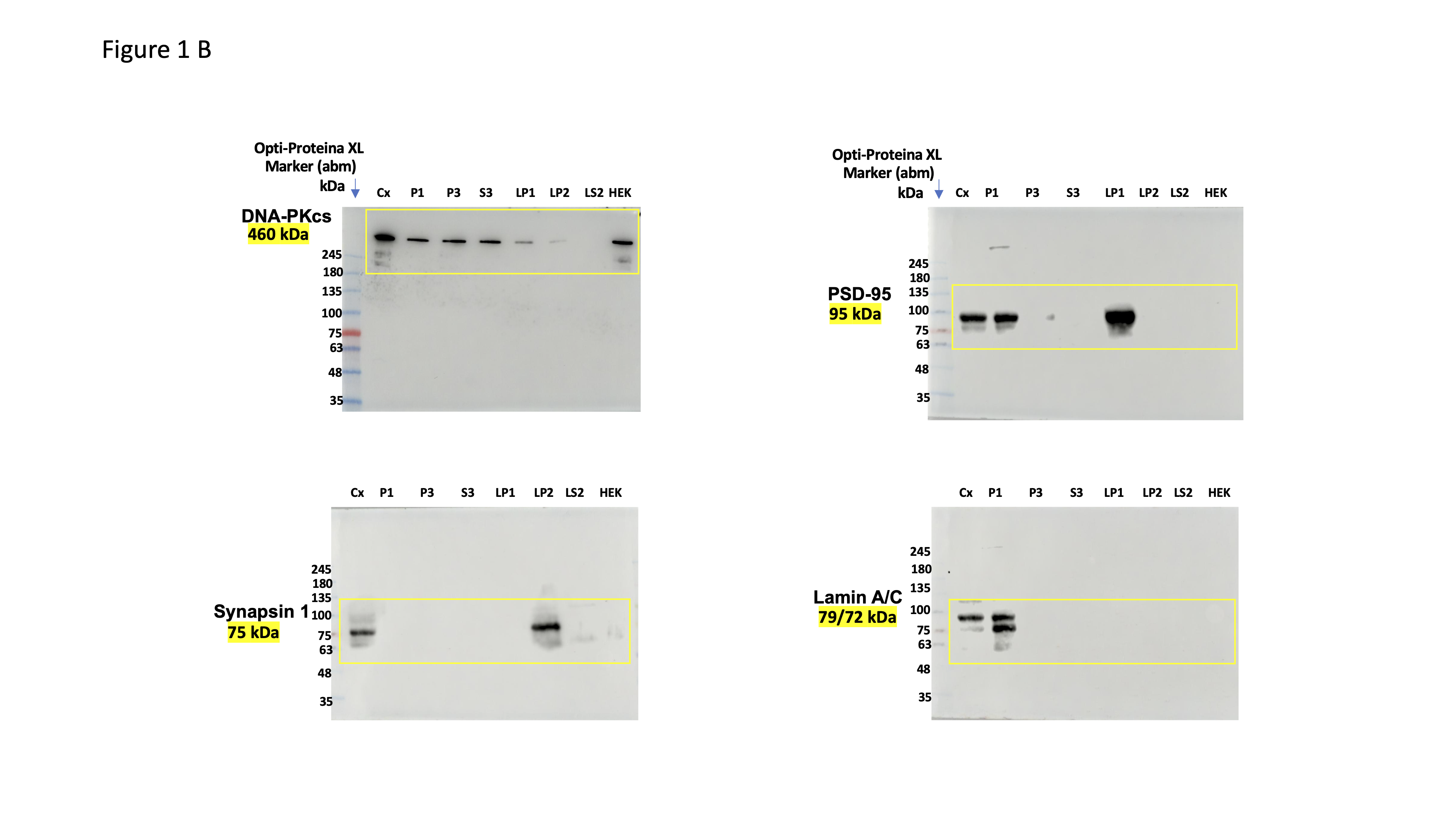

Supplement: Supplementary file 4 — Source data Fig. 1 [file 44319_2024_198_MOESM4_ESM.zip › Figure 1/Figure 1B/Figure 1B.tiff]

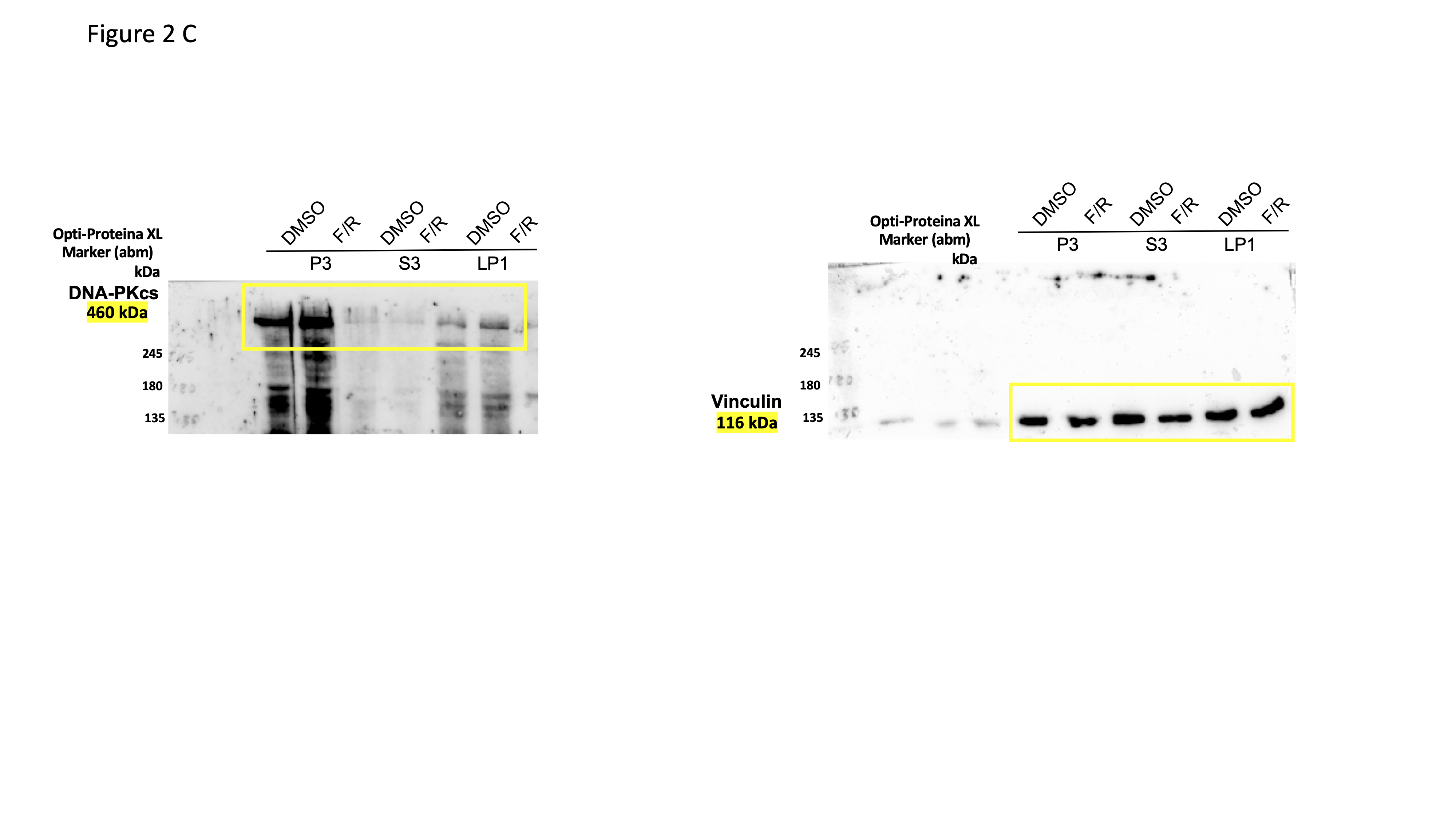

Supplement: Supplementary file 5 — Source data Fig. 2 [file 44319_2024_198_MOESM5_ESM.zip › Figure 2/Figure 2C/Figure 2C.tiff]

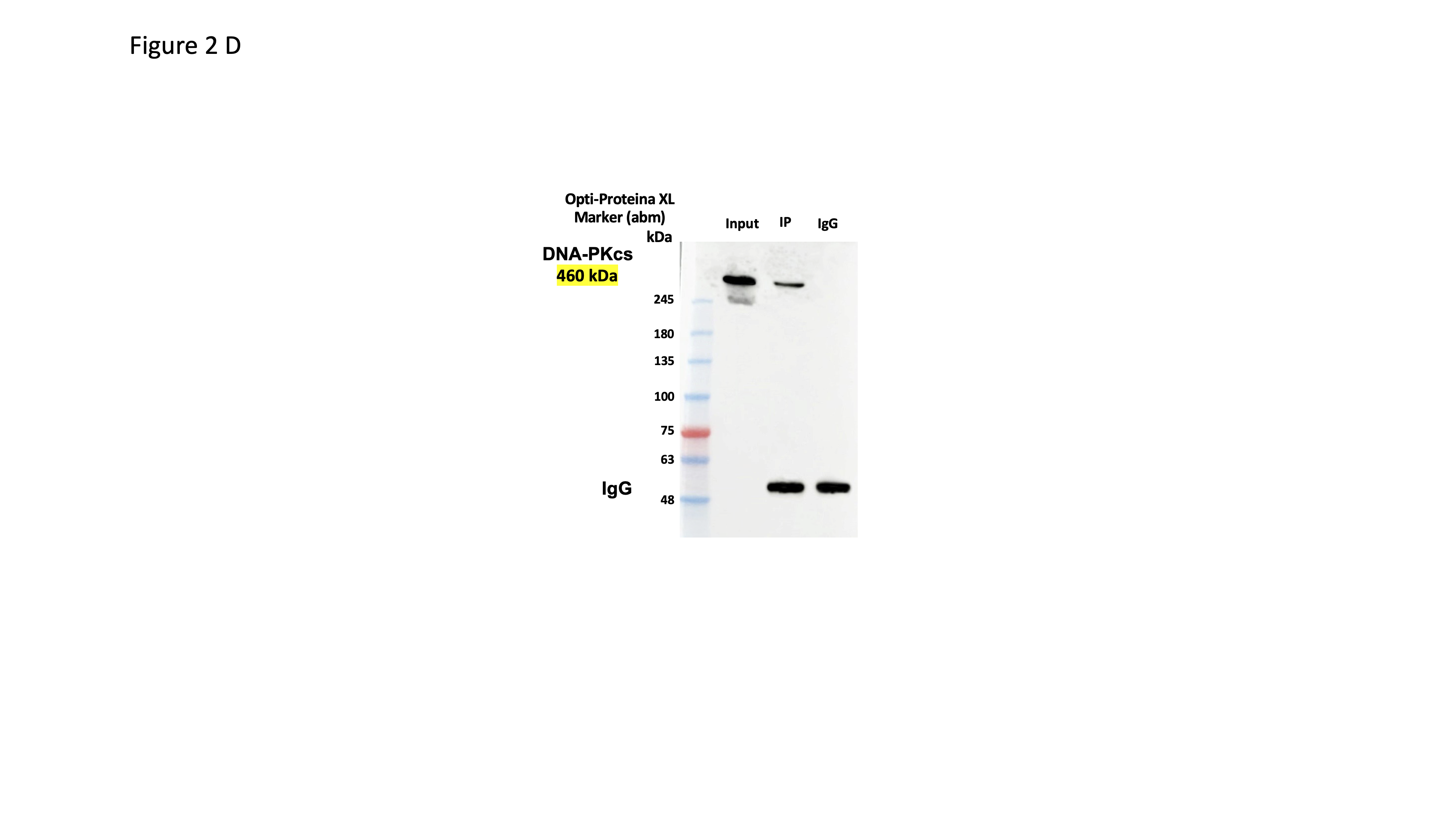

Supplement: Supplementary file 5 — Source data Fig. 2 [file 44319_2024_198_MOESM5_ESM.zip › Figure 2/Figure 2D/Figure 2D.tiff]

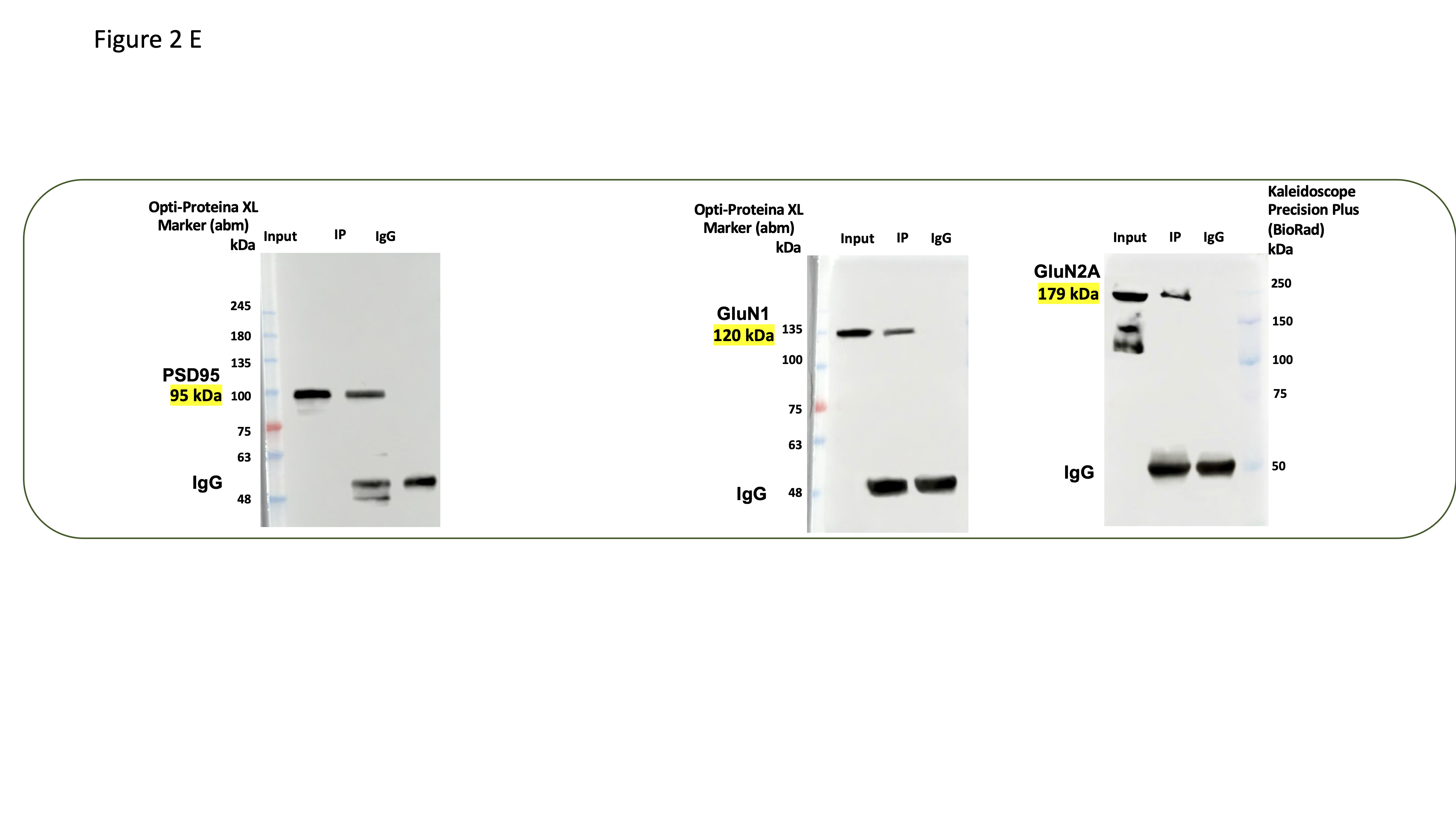

Supplement: Supplementary file 5 — Source data Fig. 2 [file 44319_2024_198_MOESM5_ESM.zip › Figure 2/Figure 2E/Figure 2E.tiff]

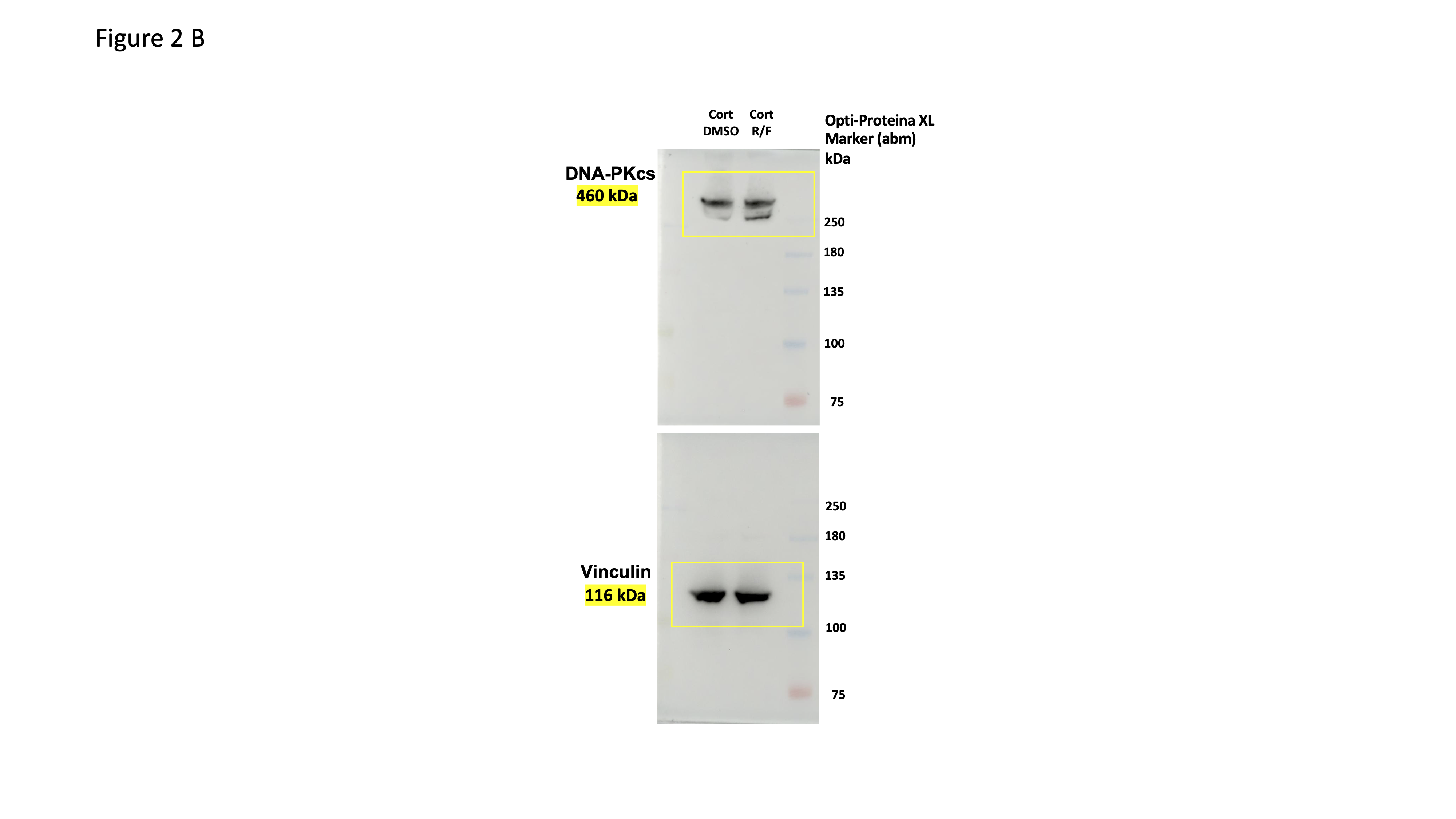

Supplement: Supplementary file 5 — Source data Fig. 2 [file 44319_2024_198_MOESM5_ESM.zip › Figure 2/Figure 2B/Figure 2B.tiff]

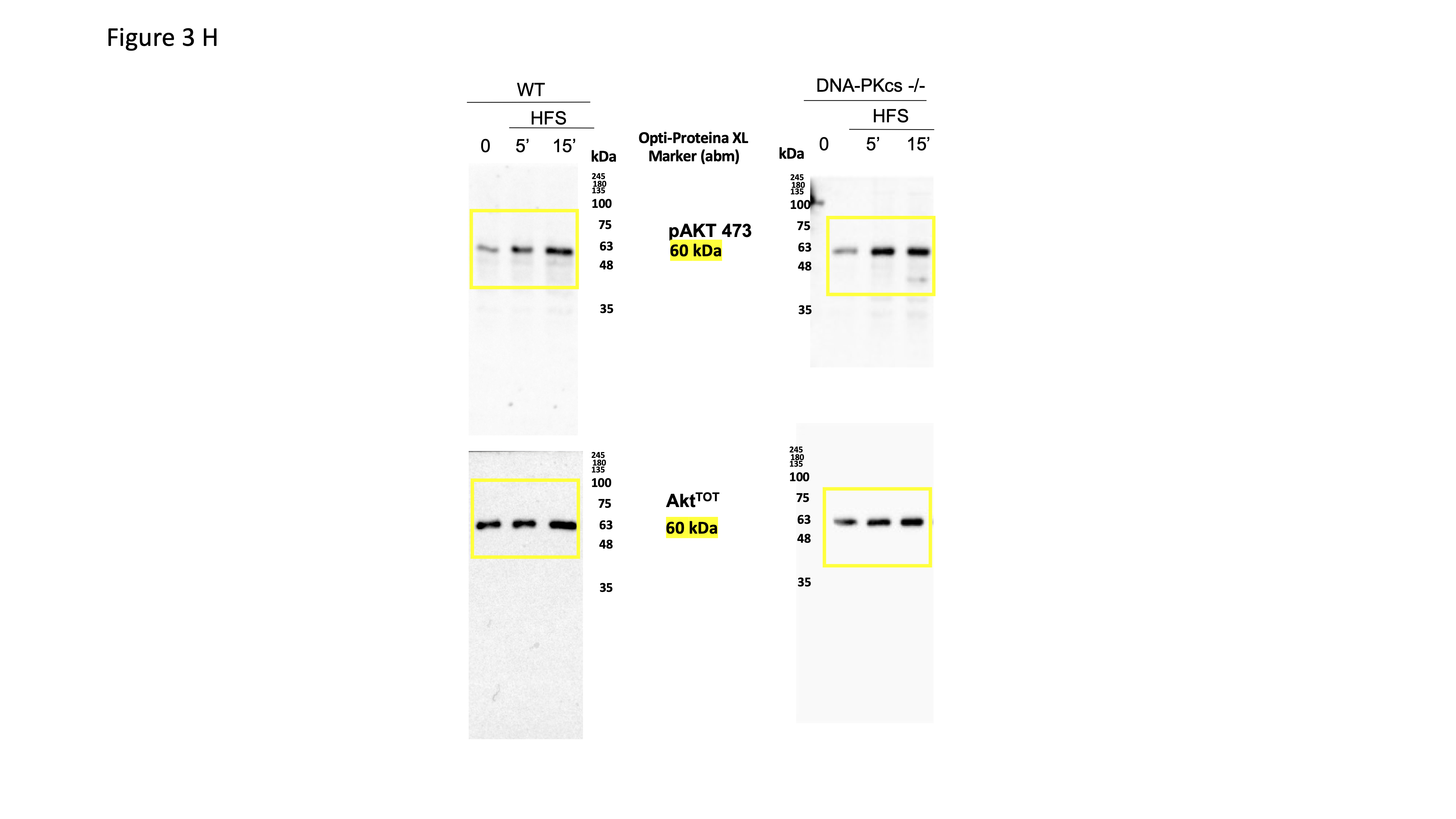

Supplement: Supplementary file 6 — Source data Fig. 3 [file 44319_2024_198_MOESM6_ESM.zip › Figure 3/Figure 3H/Figure 3H - Akt.tiff]

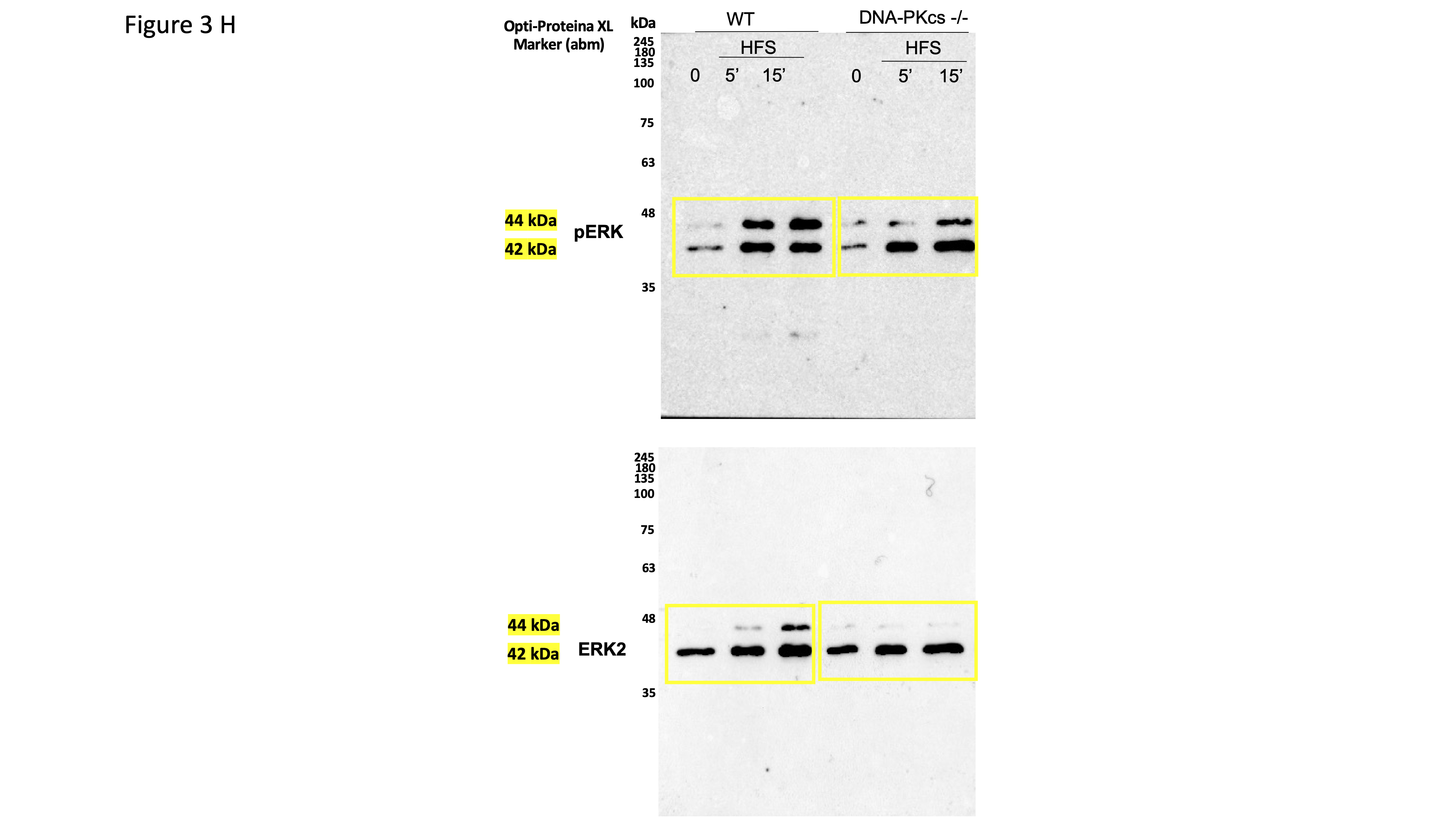

Supplement: Supplementary file 6 — Source data Fig. 3 [file 44319_2024_198_MOESM6_ESM.zip › Figure 3/Figure 3H/Figure 3H - ERK.tiff]

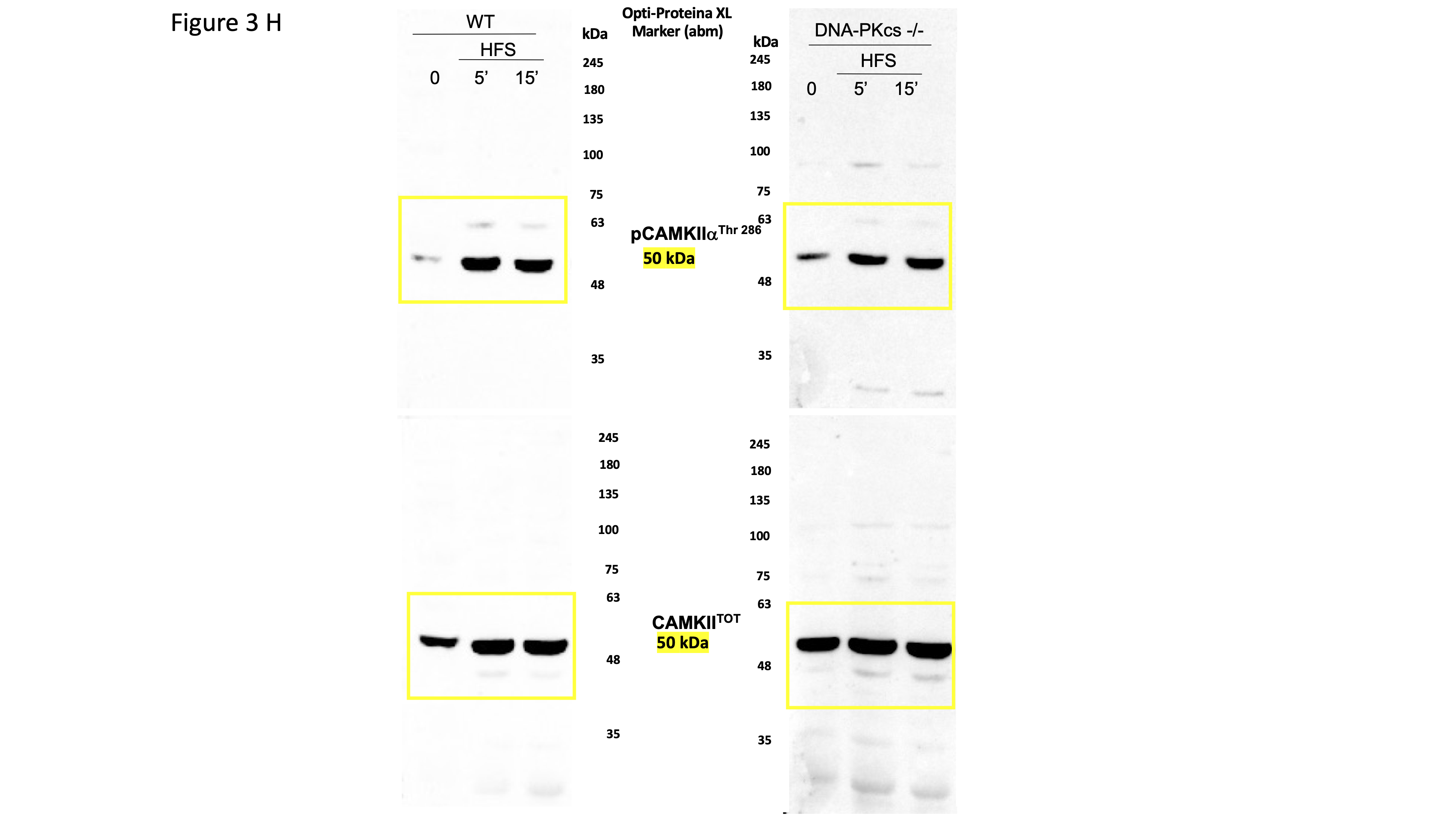

Supplement: Supplementary file 6 — Source data Fig. 3 [file 44319_2024_198_MOESM6_ESM.zip › Figure 3/Figure 3H/Figure 3H - CAMKII.tiff]

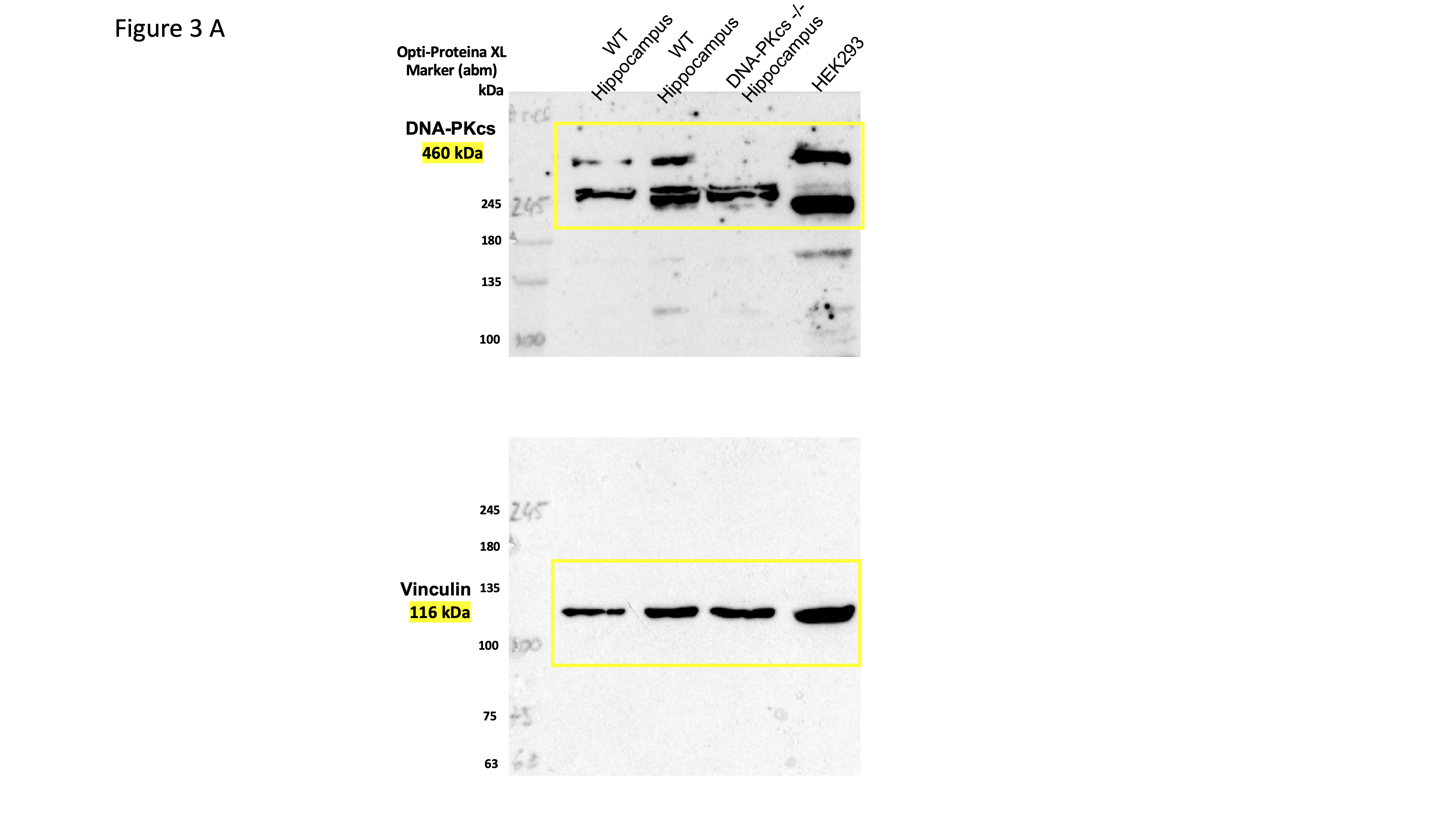

Supplement: Supplementary file 6 — Source data Fig. 3 [file 44319_2024_198_MOESM6_ESM.zip › Figure 3/Figure 3A/Figure 3A.tiff]

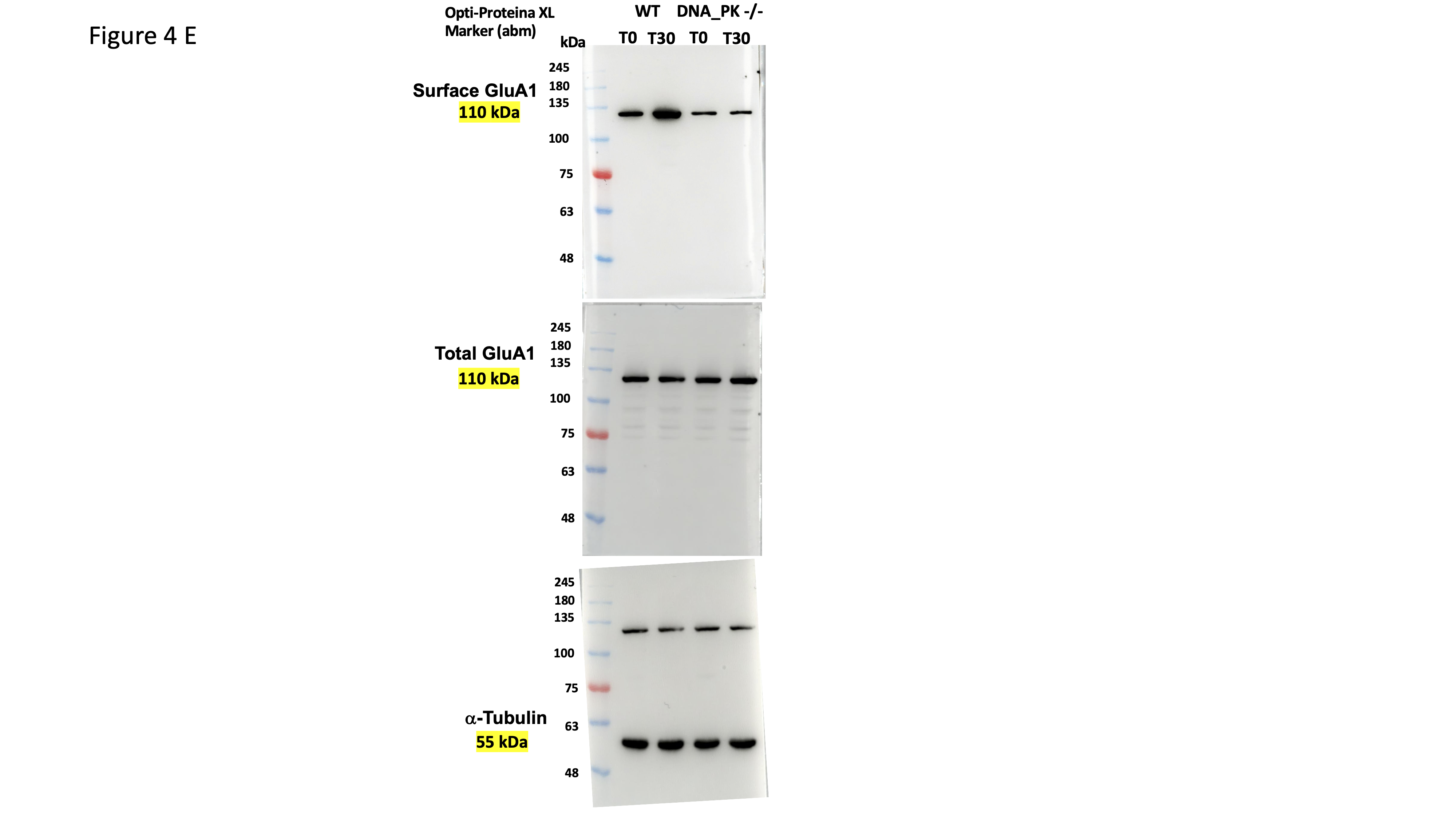

Supplement: Supplementary file 7 — Source data Fig. 4 [file 44319_2024_198_MOESM7_ESM.zip › Figure 4/Figure 4E/Figure 4E.tiff]

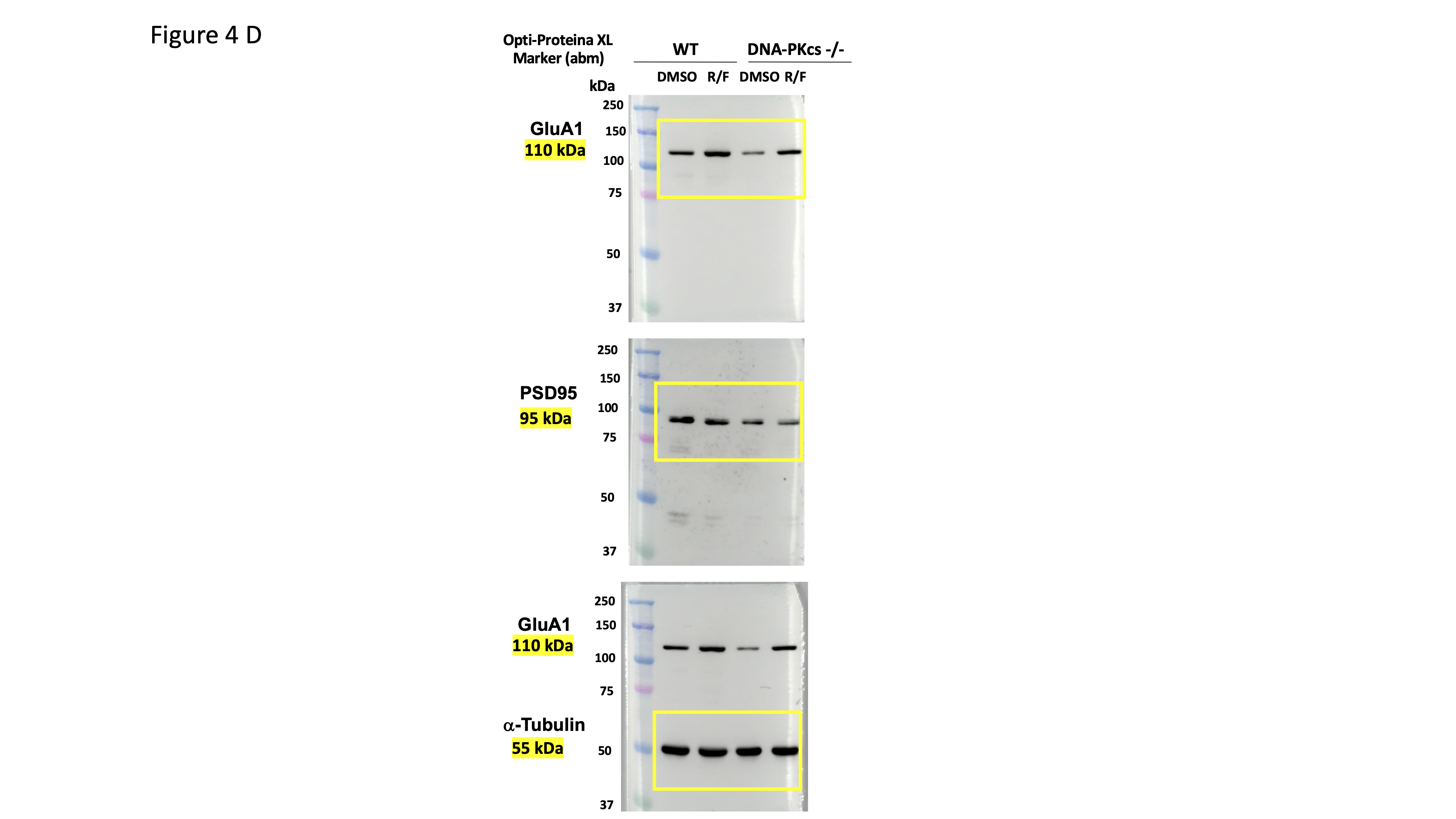

Supplement: Supplementary file 7 — Source data Fig. 4 [file 44319_2024_198_MOESM7_ESM.zip › Figure 4/Figure 4D/Figure 4D.tiff]

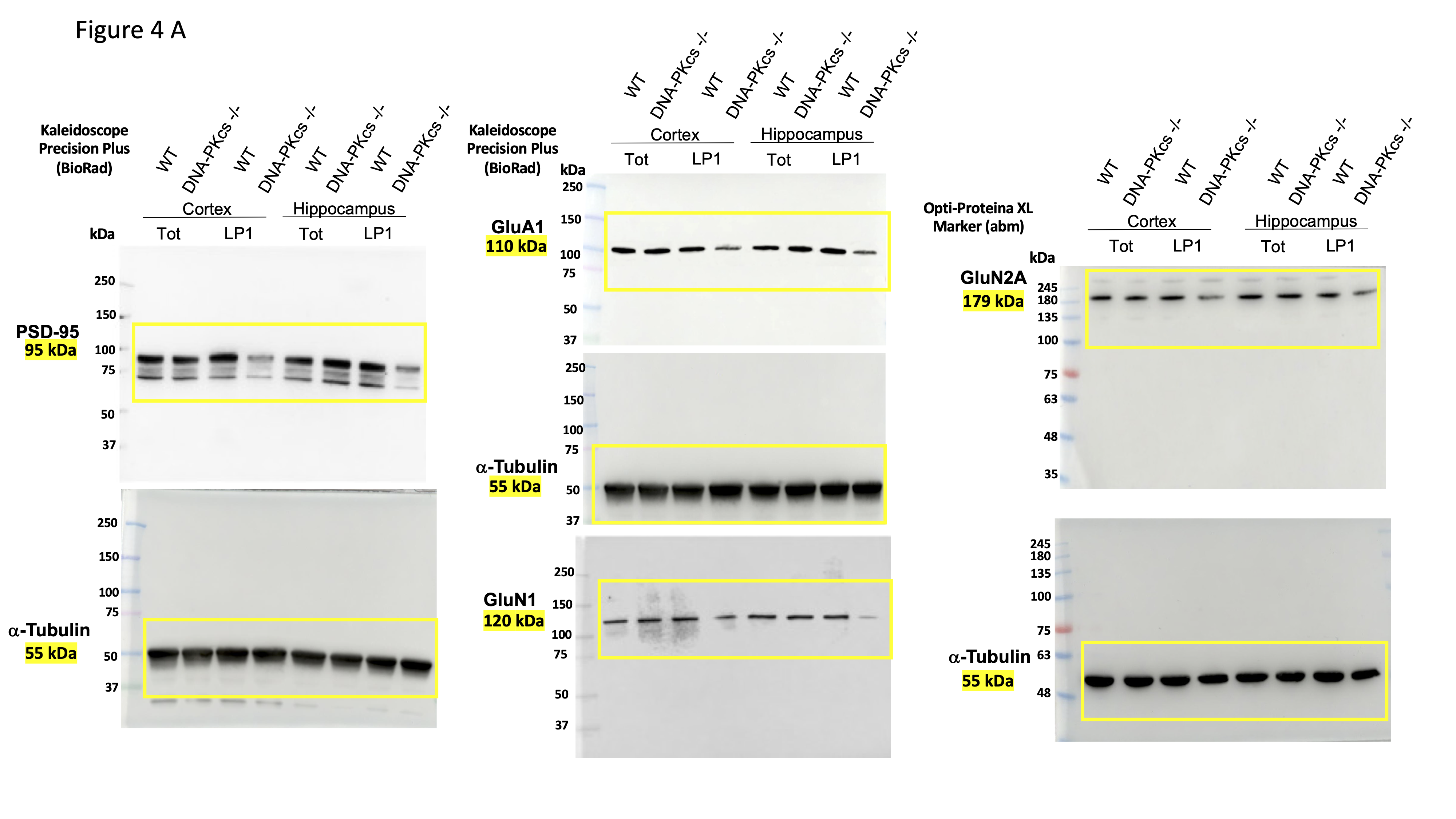

Supplement: Supplementary file 7 — Source data Fig. 4 [file 44319_2024_198_MOESM7_ESM.zip › Figure 4/Figure 4A/Figure 4A.tiff]

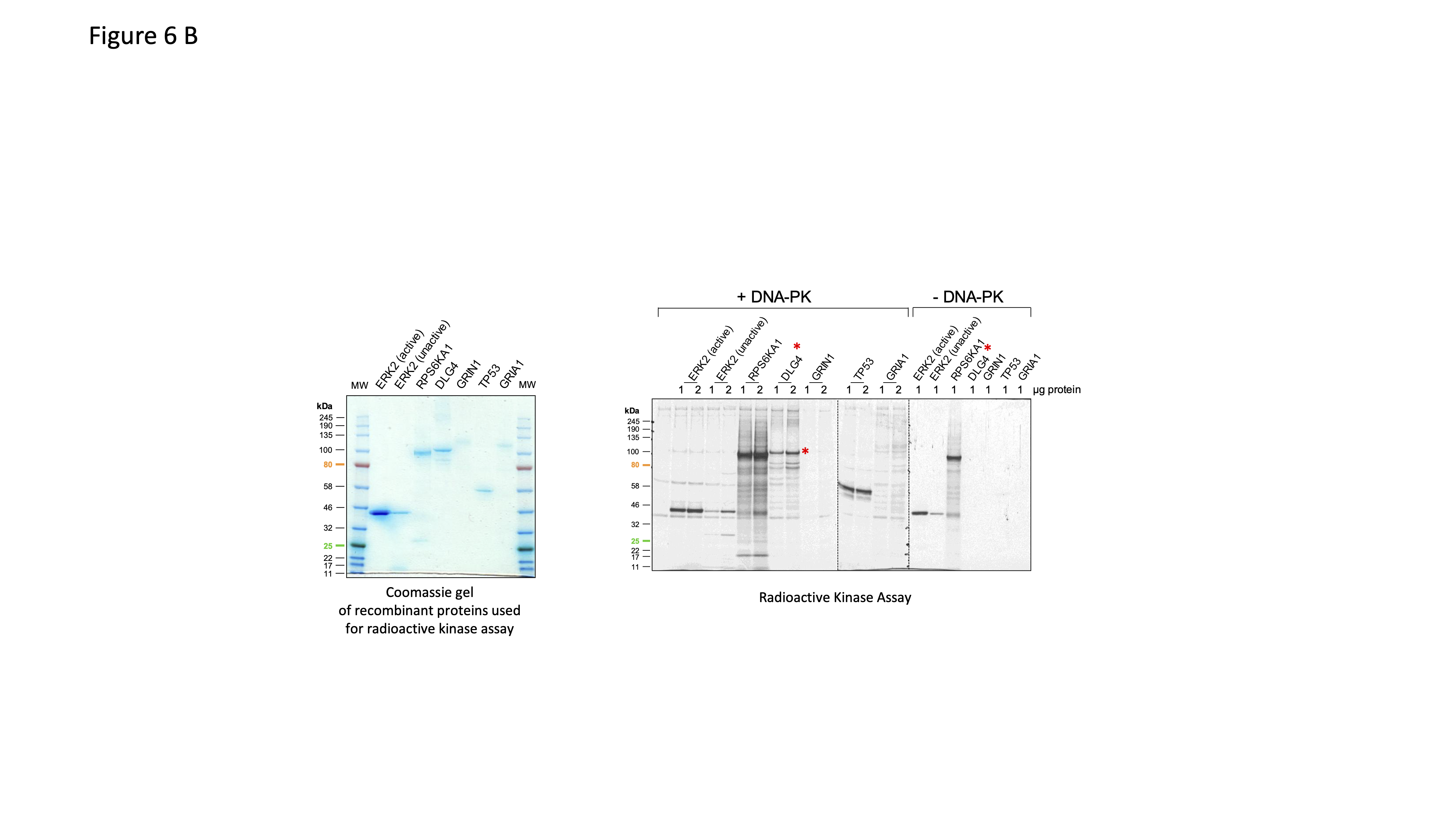

Supplement: Supplementary file 9 — Source data Fig. 6 [file 44319_2024_198_MOESM9_ESM.zip › Figure 6/Figure 6B/Figure 6B.tiff]

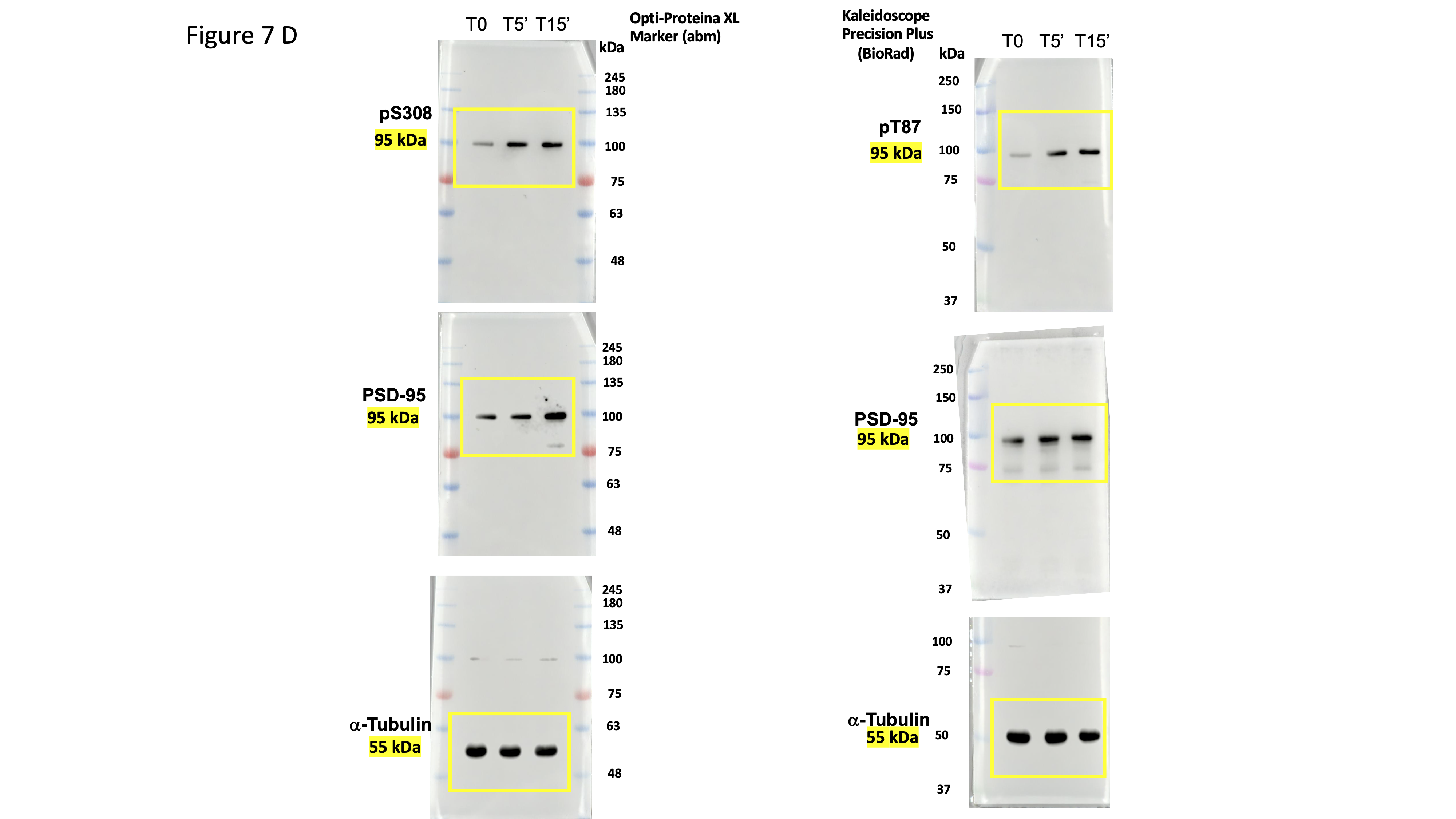

Supplement: Supplementary file 10 — Source data Fig. 7 [file 44319_2024_198_MOESM10_ESM.zip › Figure 7/Figure 7D/Figure 7D.tiff]

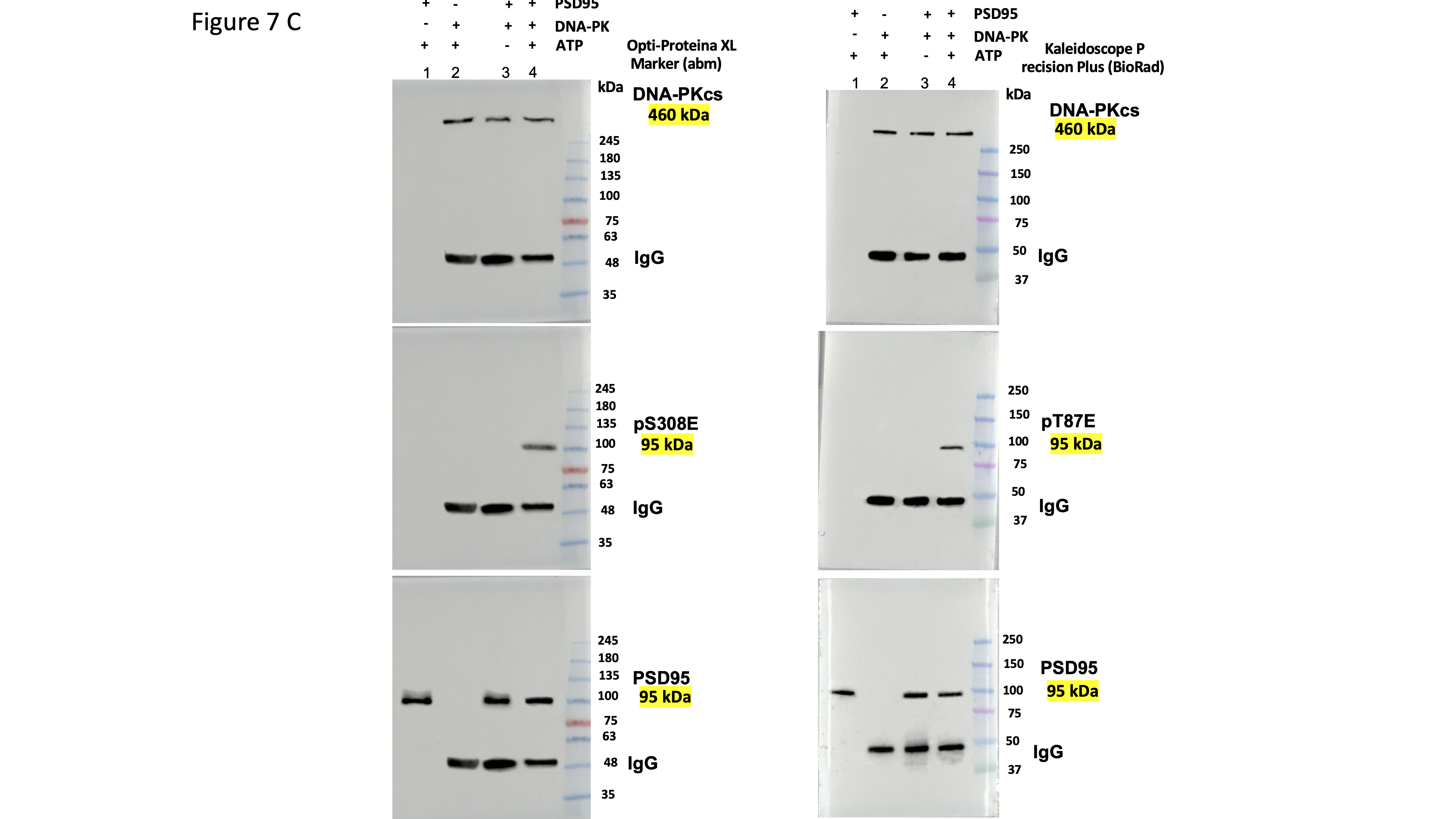

Supplement: Supplementary file 10 — Source data Fig. 7 [file 44319_2024_198_MOESM10_ESM.zip › Figure 7/Figure 7C/Figure 7C.tiff]

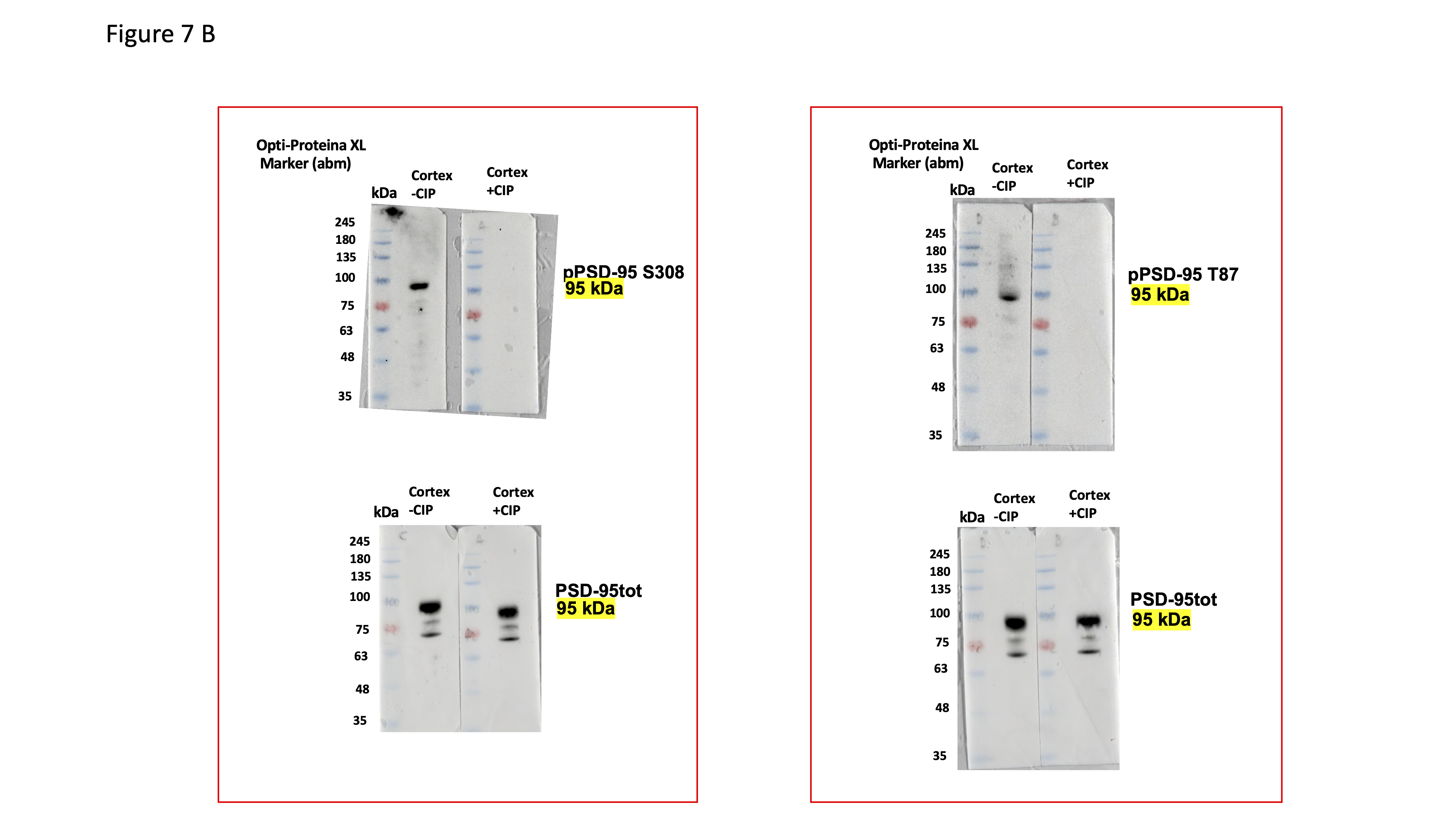

Supplement: Supplementary file 10 — Source data Fig. 7 [file 44319_2024_198_MOESM10_ESM.zip › Figure 7/Figure 7B/Figure 7B.tiff]

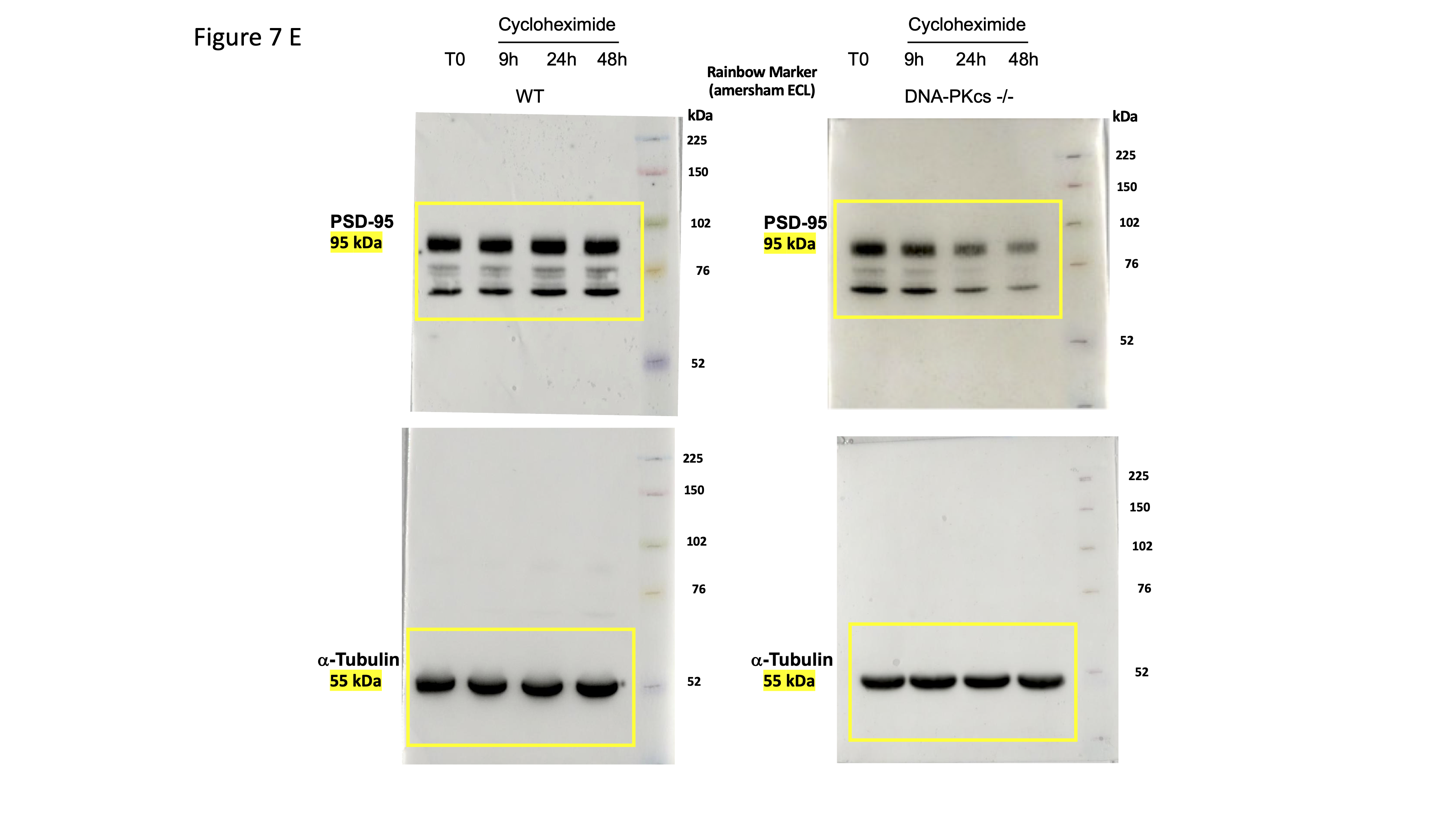

Supplement: Supplementary file 10 — Source data Fig. 7 [file 44319_2024_198_MOESM10_ESM.zip › Figure 7/Figure 7E/Figure 7E.tiff]

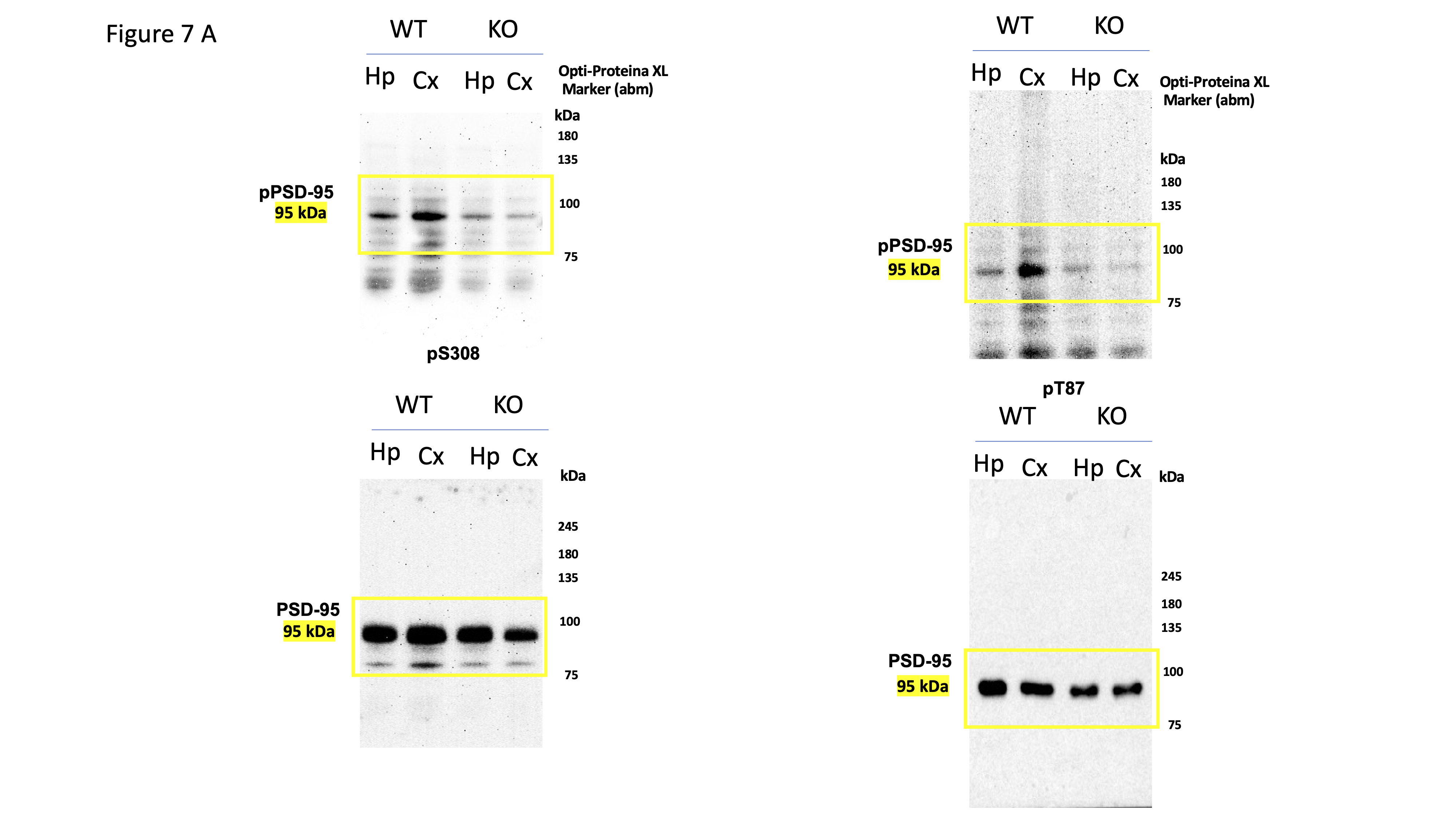

Supplement: Supplementary file 10 — Source data Fig. 7 [file 44319_2024_198_MOESM10_ESM.zip › Figure 7/Figure 7A/Figure 7A.tiff]

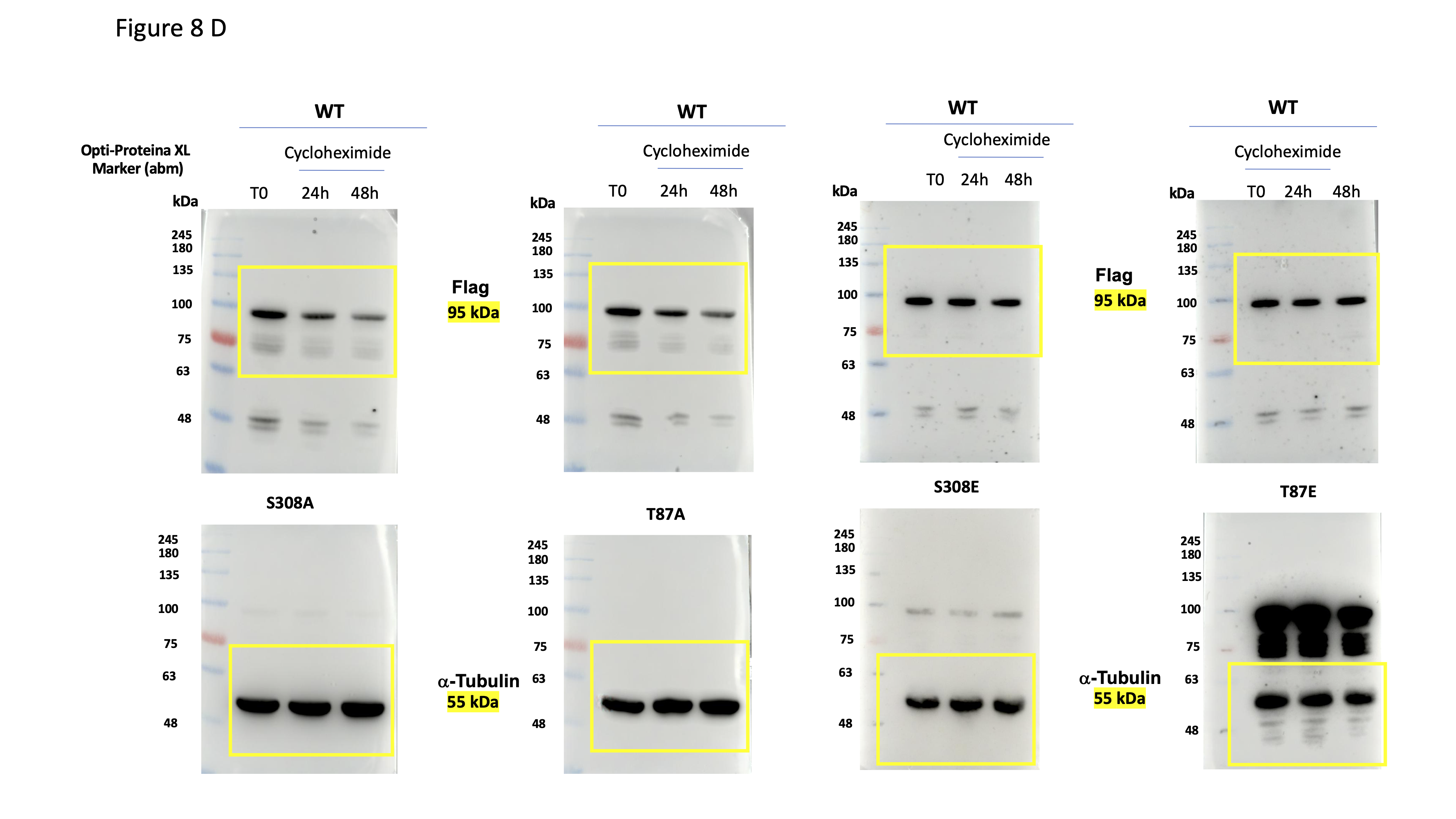

Supplement: Supplementary file 11 — Source data Fig. 8 [file 44319_2024_198_MOESM11_ESM.zip › Figure 8/Figure 8D/Figure 8D.tiff]

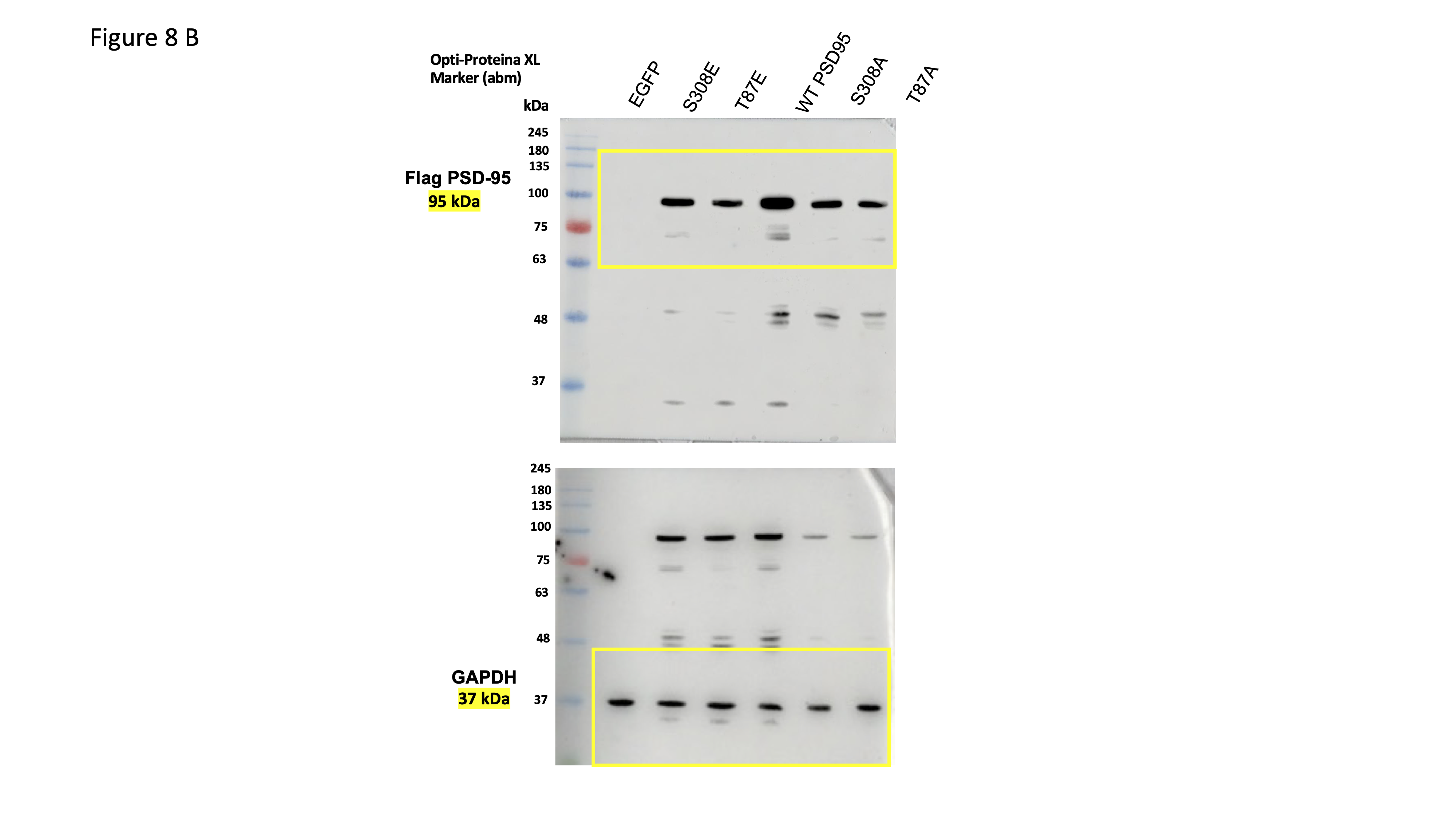

Supplement: Supplementary file 11 — Source data Fig. 8 [file 44319_2024_198_MOESM11_ESM.zip › Figure 8/Figure 8B/Figure 8B.tiff]

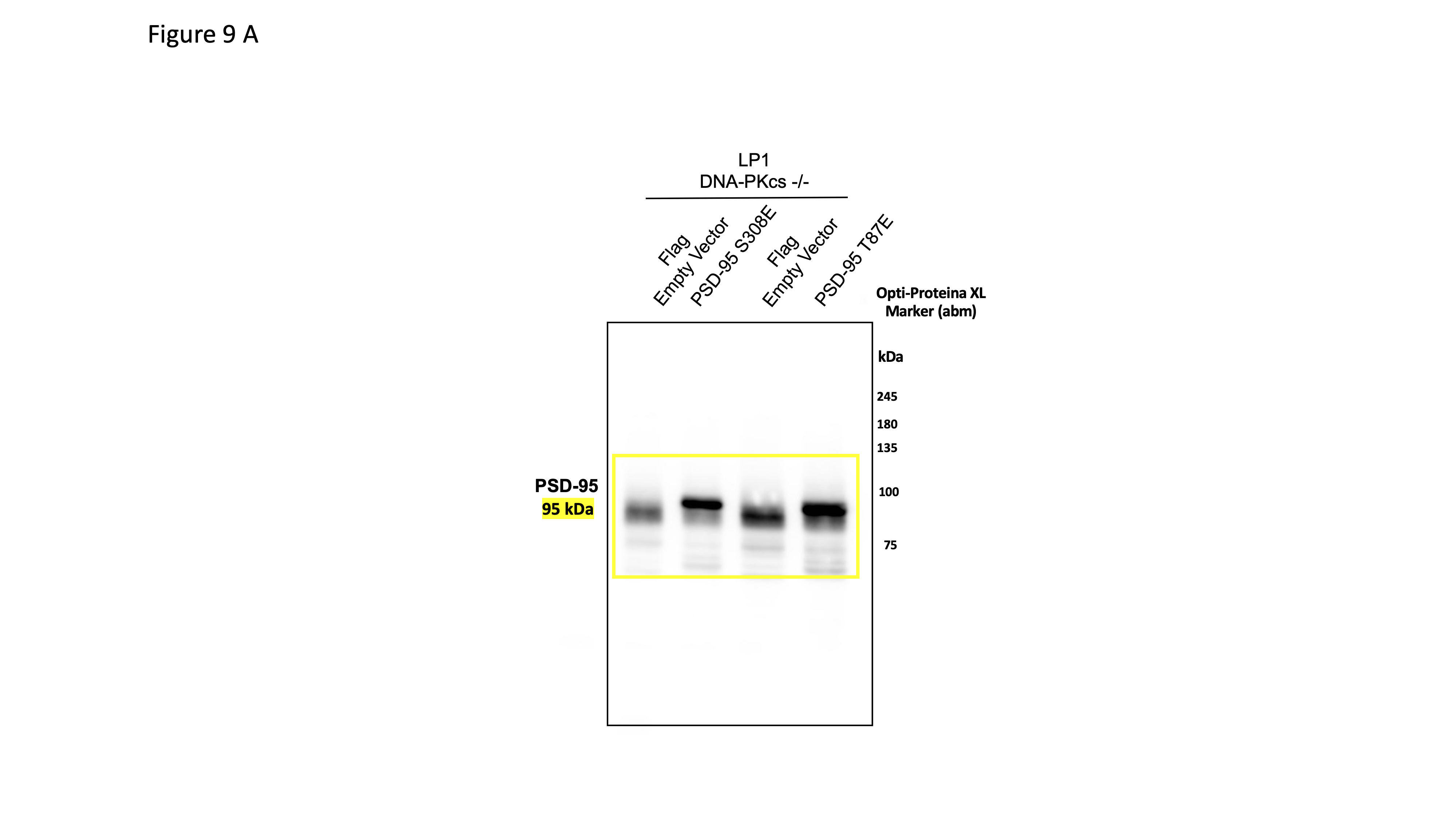

Supplement: Supplementary file 12 — Source data Fig. 9 [file 44319_2024_198_MOESM12_ESM.zip › Figure 9/Figure 9A/Figure 9A.tiff]

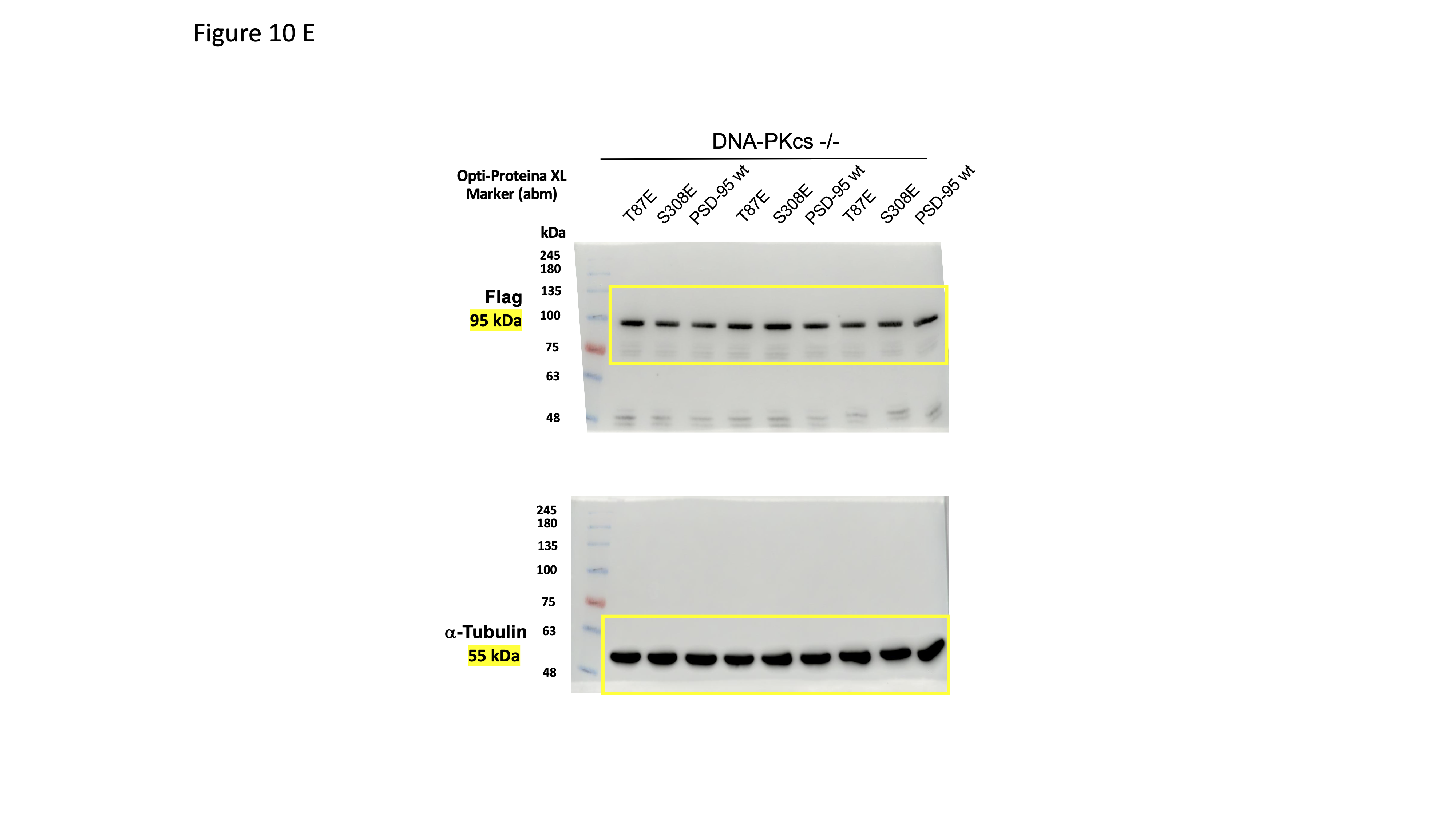

Supplement: Supplementary file 13 — Source data Fig. 10 [file 44319_2024_198_MOESM13_ESM.zip › Figure 10/Figure 10E/Figure 10E.tiff]

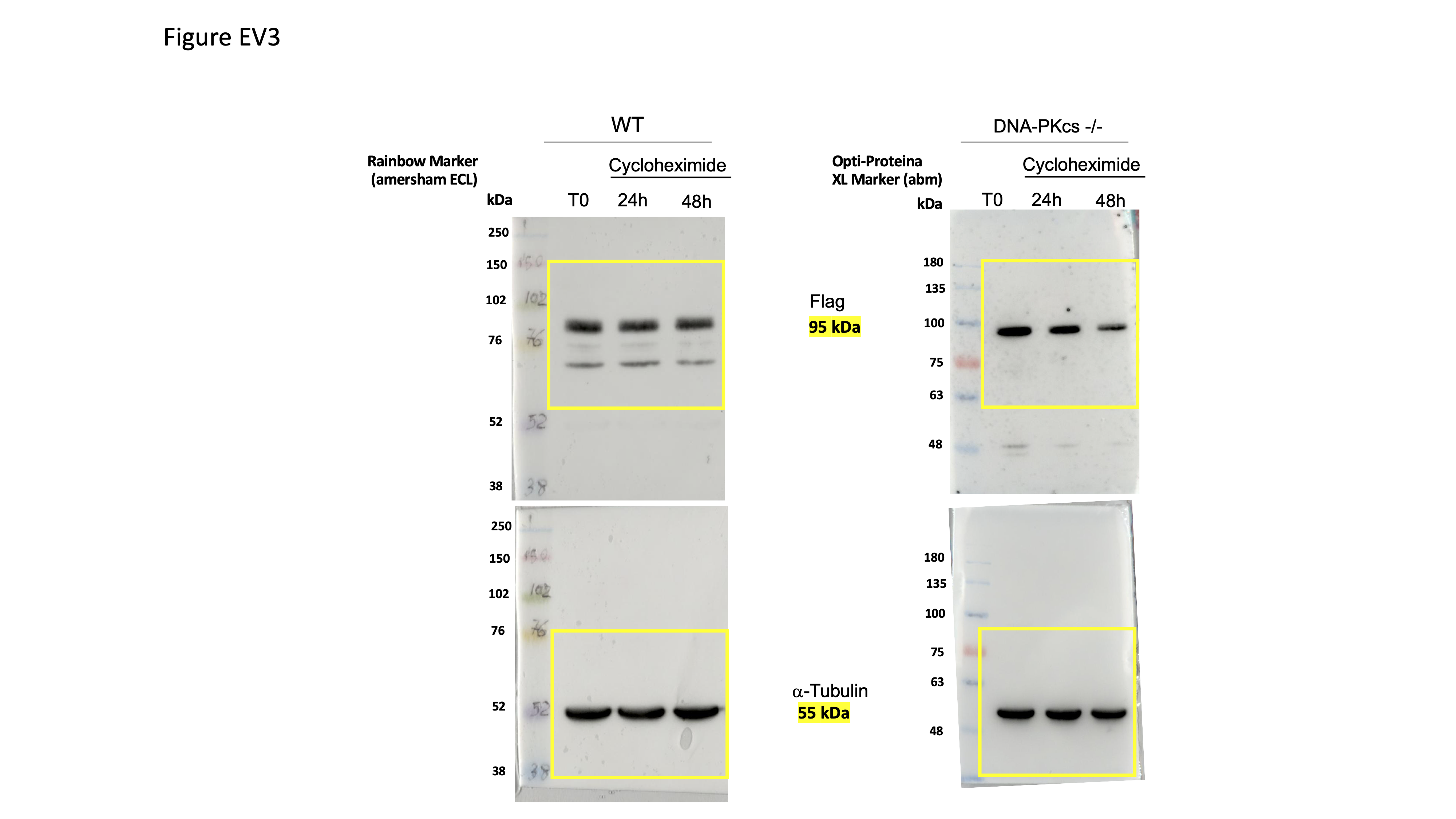

Supplement: Supplementary file 14 — Figure EV3 Source Data [file 44319_2024_198_MOESM14_ESM.zip › Figure EV3/Figure EV3.tiff]
