# Supplementary material for: The DNA repair protein DNA-PKcs modulates synaptic plasticity via PSD-95 phosphorylation and stability
Source: EMBO Rep. 2024 Jul 31;25(8):27. doi: 10.1038/s44319-024-00198-3 (PMC11315936; doi:10.1038/s44319-024-00198-3)
Supplement: Supplementary file 15 — Expanded View Figures [file 44319_2024_198_MOESM15_ESM.pdf]

Expanded View Figures

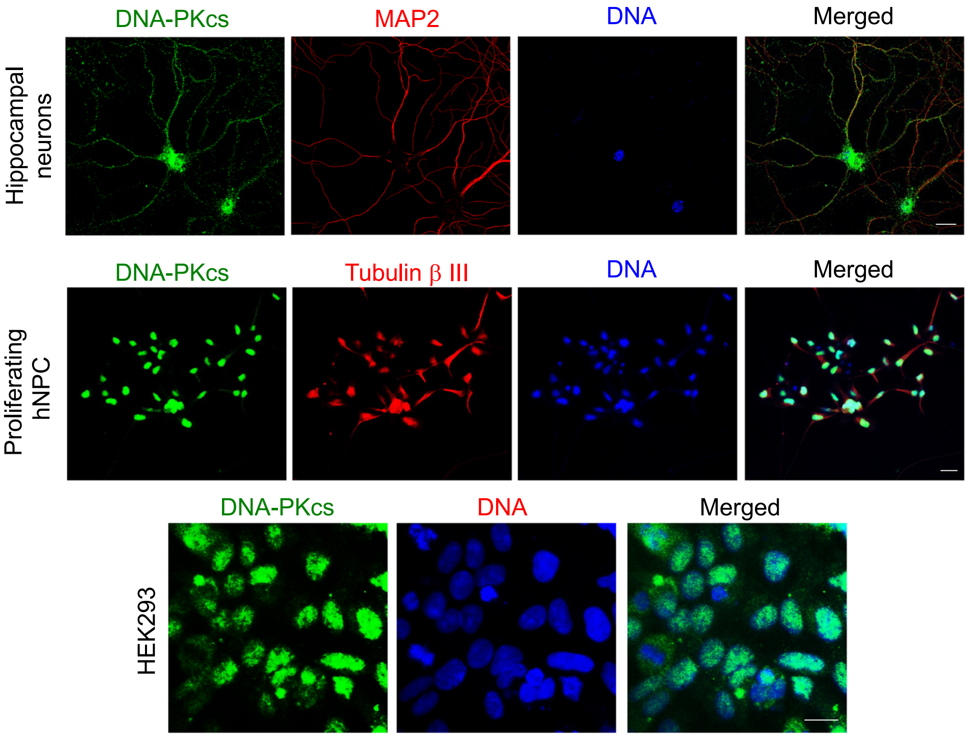

Figure EV1. DNA-PKcs has a lower nuclear staining in post-mitotic neurons as compared with proliferating cells.

Representative immunofluorescence images showing the distribution of DNA-PKcs (green) in post-mitotic mouse hippocampal neurons as compared with proliferating human neural progenitor cells (NPCs) and HEK293 cells. In proliferating cells, DNA-PKcs appear mainly concentrated in the nucleus, whereas in neurons, it is abundant along dendrites showing a punctate staining and lower nuclear labeling. Scale Bar 10 μm.

© The Author(s)

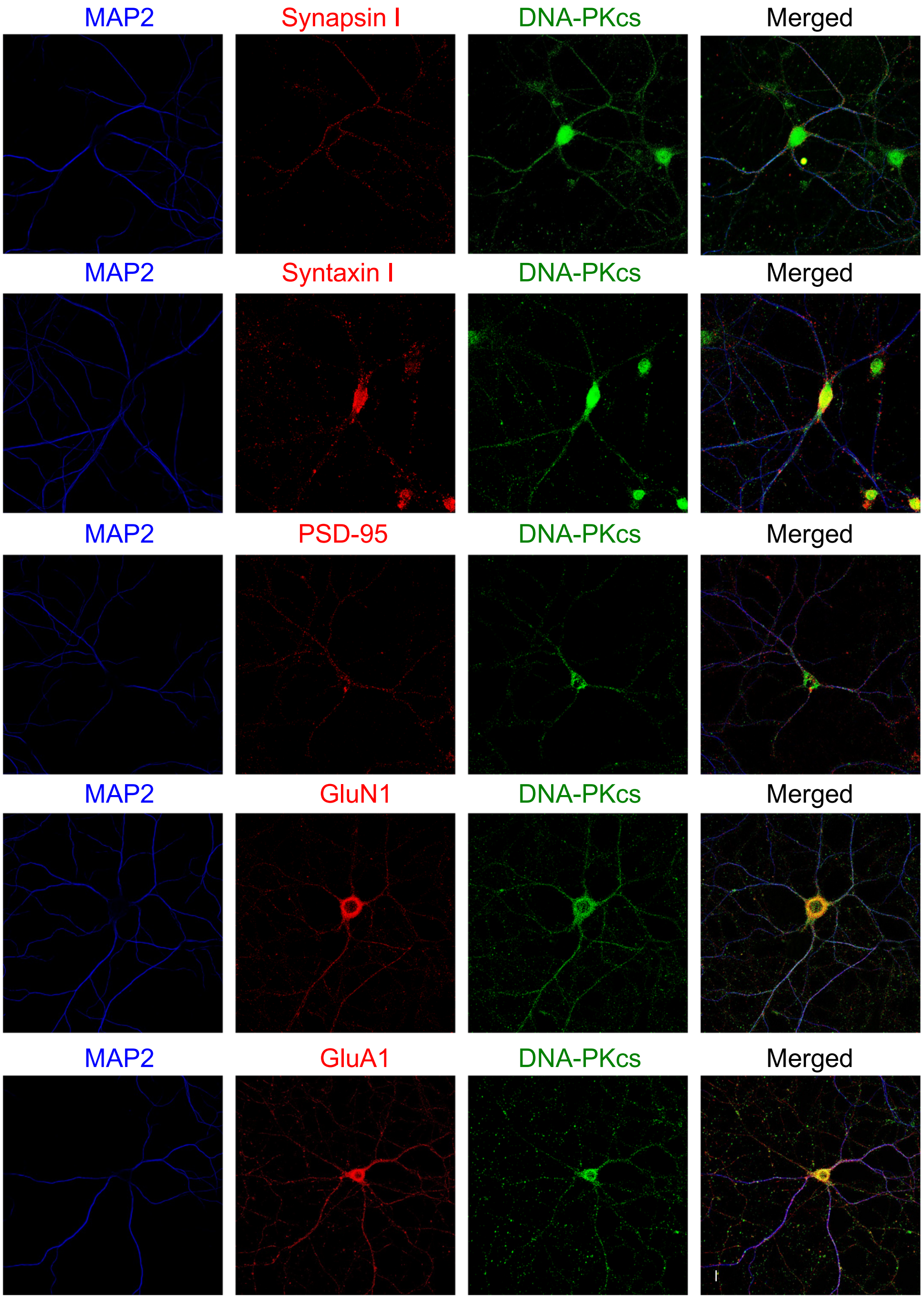

Figure EV2. DNA-PKcs shows a synaptic distribution in cortical neurons.

Representative triple immunofluorescence THUNDER images of mouse primary cortical neurons (DIV 21) labeled with the anti-DNA-PKcs antibody (green channel), the synaptic markers: Synapsin I, Syntaxin I, PSD-95, GluN1, and GluA1 (red channel), one at a time, and the neuronal marker MAP2 (blue). Single-channel images are provided to better show the localization of each synaptic marker and the distribution of DNA-PKcs similar to the synaptic proteins. Scale Bar 5 µm.

 © The Author(s)

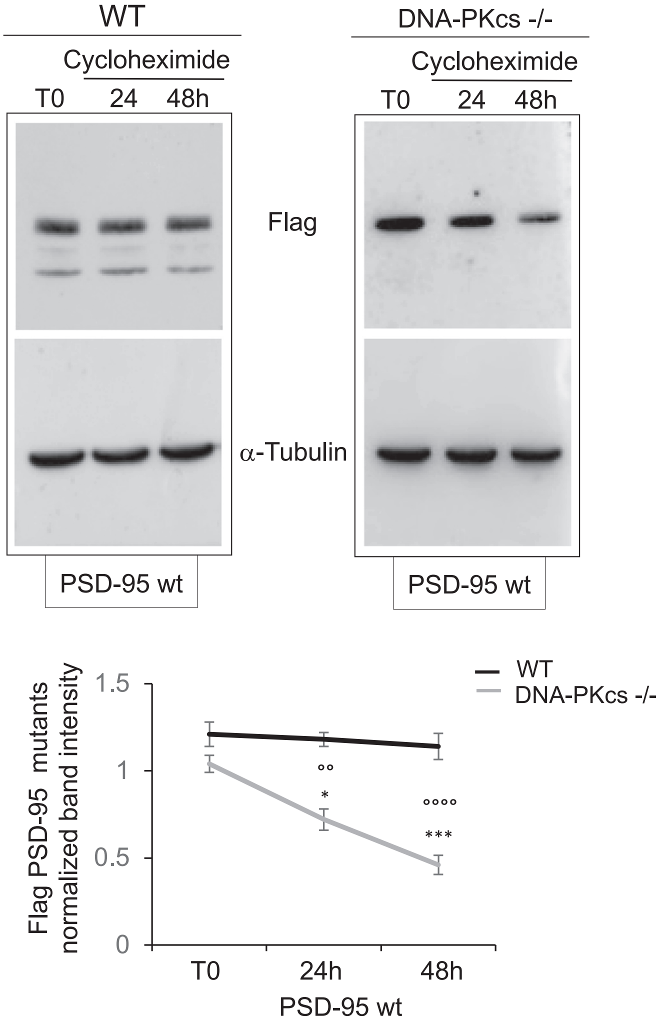

Figure EV3. PSD-95 wt over-expressed protein is less stable in the absence of DNA-PKcs kinase activity.

Representative Western blots of protein extracts from cortical neurons of WT and DNA-PKcs −/− mice show that PSD-95 wt protein, over-expressed in WT neurons, remains stable after cycloheximide treatment up to 48 h, whereas it decreases over time when over-expressed in DNA-PKcs −/− neurons. Values in the plot represent the quantification of PSD-95 wt protein levels over time following cycloheximide treatment normalized to α-Tubulin. (means ± SEM; $n = 3$). Statistics by two-way ANOVA followed by Bonferroni post hoc analysis. *$p < 0.05$ DNA-PKcs 24 h vs DNA-PKcs T0, ***$p < 0.001$ DNA-PKcs 48 h vs DNA-PKcs T0, °°$p < 0.005$ DNA-PKcs 24 h vs WT 24 h, °°°°$p < 0.0001$ DNA-PKcs −/− 48 h vs WT 48 h. Source data are available online for this figure.
